# Nitric oxide synthase-guided genome mining identifies a cytochrome P450 enzyme for olefin nitration in bacterial specialized metabolism

**DOI:** 10.1016/j.synbio.2024.01.005

**Published:** 2024-01-17

**Authors:** Hu Li, Wei Li, Kaihui Song, Yu Liu, Guiyun Zhao, Yi-Ling Du

**Affiliations:** a Polytechnic Institute, Zhejiang University, Hangzhou, 310022, China; b Department of Microbiology, Zhejiang University School of Medicine, Hangzhou, 310058, China; c College of Life Sciences, Zhejiang University, 310058, Hangzhou, China

**Keywords:** Nitric oxide, Genome mining, Biosynthesis, Nitration, Cytochrome, P450

## Abstract

The biological signaling molecule nitric oxide (NO) has recently emerged as a metabolic precursor for the creation of microbial natural products with diversified structures and biological activities. Within the biosynthetic gene clusters (BGCs) of these compounds, genes associated with NO production pathways have been pinpointed. In this study, we employ a nitric oxide synthase (NOS)-guided genome mining strategy for the targeted discovery of NO-derived bacterial natural products and NO-utilizing biocatalysts. We show that a conserved NOS-containing BGC, distributed across several actinobacterial genomes, is responsible for the biosynthesis of lajollamycin, a unique nitro-tetraene-containing antibiotic whose biosynthetic mechanism remains elusive. Through a combination of in vivo and in vitro studies, we unveil the first cytochrome P450 enzyme capable of catalyzing olefin nitration in natural product biosynthesis. These results not only expand the current knowledge about biosynthetic nitration processes but also offer an efficient way for targeted identification of NO-utilizing metabolic pathways and novel nitrating biocatalysts.

## 1 Introduction

Nitric oxide (NO) plays multifaceted roles in biology and regulates a variety of physiological processes including metabolism, neurotransmission, immunity, and cardiovascular system [[1], [2], [3]]. Beyond its role as a signaling molecule, there is increasing evidence indicating that NO can also serve as a metabolic precursor to various specialized metabolites [4]. Among the established biological routes to NO, nitric oxide synthase (NOS)-mediated pathways are widely distributed across different biological kingdoms [5,6]. Despite the distinctive domain organizations between mammalian and bacterial NOSs, both enzymes catalyze oxidations of l-arginine (l-Arg), converting it to l-citrulline and NO, with *N*^G^-hydroxyl-l-arginine as an intermediate [7]. Alternatively, NO can also be generated through the reduction of inorganic nitrate and nitrite by nitrate/nitrite reductase, which is also known as the nitrate-nitrite-NO pathway [8,9].

**Fig. 1:**
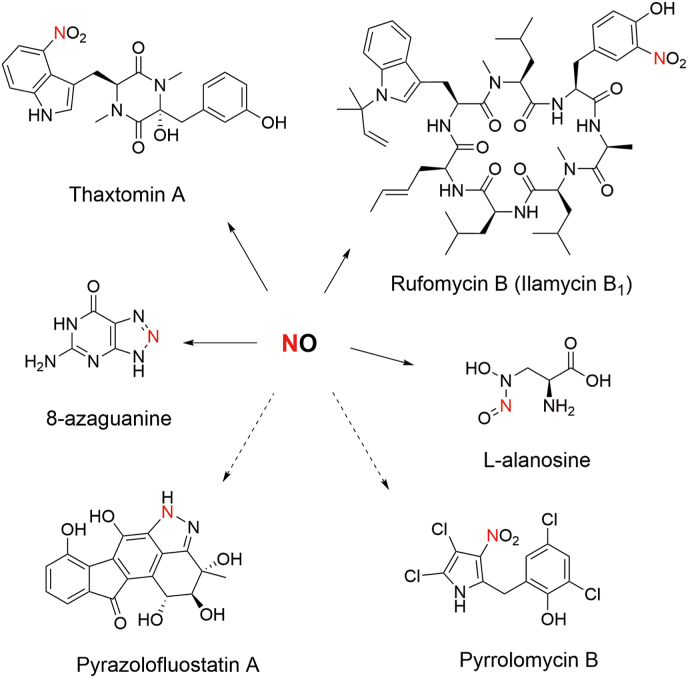
Microbial natural products with nitric oxide as a (potential) metabolic precursor. Note: NO have been experimentally confirmed to be a precursor for thaxtomin A, rufomycin B/ilamycin B_1_, 8-azaguanine, 2-azaphypoxnathine and alanosine, while NO biosynthetic genes are present in the biosynthetic gene clusters of pyrazolofluostatin A and pyrrolomycin B (dash line).

The initial discovery of nitric oxide (NO) involvement in natural product biosynthesis emerged from the investigation into the thaxtomin phytotoxin biosynthetic pathway found in *Streptomyces* strains (Fig. 1) [7]. It was found that the biosynthetic gene cluster (BGC) of thaxtomin (*txt* BGC) encodes a bacterial NOS (TxtD), which provides NO as the nitrogen atom donor for the nitro group present in the structure of thaxtomin A. Subsequently, a cytochrome P450 enzyme (TxtE) was shown to catalyze tryptophan nitration using NO and O_2_ [10]. Similar aromatic nitration mediated by a NOS/P450 pair (RufN/RufO or IlaM/IlaN) was also found in the biosynthesis of rufomycins/ilamycins [[11], [12], [13], [14]]. In addition, we recently showed that the N8 atom in the unique 1,2,3-triazole heterocycle of the antiviral agent 8-azaguanine derives from NO, which is synthesized by a NOS (PtnF) [15]. Moreover, fungal NOS was demonstrated in contributing to 1,2,3-triazine formation in the biosynthesis of plant-growth regulator 2-azahypoxanthine [16]. Notably, the BGC of pyrazo-containing polyketide pyrazolofluostatins also harbor a putative NOS gene (*flsN1*), indicating the involvement of NOS in its biosynthetic pathway [17]. In addition to the NOS-mediated pathway, NO production in the biosynthesis of bacterial specialized metabolites could also occur through a process analogous to assimilatory nitrate/nitrite reduction. Genes encoding homologues of nitrate-nitrite reductase components have been identified in the BGCs of pyrrolomycin (*pyr18/pyr19*) and l-alanosine (*alnP*/*alnQ*/*alnR*). These genes were suggested or linked to the formation of the nitro group in pyrrolomycin B or the diazeniumdioate moiety in l-alanosine, respectively [[18], [19], [20]].

The observation that NO is utilized as a metabolic precursor for microbial natural products with distinct structures and bioactivities, indicates the potential use of NO biosynthesis gene(s) as genetic markers to discover more NO-incorporating natural products and novel NO-utilizing enzymes. In this study, we perform NOS-guided genome mining to further interrogate the role of NO in bacterial specialized metabolism. We identify a NOS-containing biosynthetic gene cluster distributed across many actinobacteria genomes, and show that this BGC encodes lajollamycin, a unique nitro-tetraene-containing antibiotic. By combining in vivo gene knockout experiments, pathway intermediate characterization, as well as in vitro biochemical assays, we demonstrated that the nitro group of lajollamycins derives from NO, and a P450 enzyme catalyzes post-assembly line olefin nitration. Although P450-catalyzed aromatic nitration has been reported previously, this is the first nitrating P450 enzyme that utilizes a linear substrate in natural product biosynthesis.

## 2 Materials and methods

### 2.1 General methods

DNA manipulations in *Escherichia coli* and *Streptomyces* strains were carried out according to standard procedures [21,22]. *Streptomyces* strains were normally maintained on MS (2% mannitol, 2% soy flour, 2% agar) agar. Apramycin (50 μg mL^−1^), kanamycin (50 μg mL^−1^), and nalidixic acid (25 μg mL^−1^) were used for selection of recombinant *E. coli* and *Streptomyces* strains. DNA primers were purchased from Tsingke Biological Technology. Reagents were purchased from Sigma-Aldrich, Thermo Fisher Scientific, New England BioLabs, Bio Basic Inc and Vazyme Biotech Co. The stock solution (100 mM) of NO donor 2-(N,N-diethylamino)-diazenolate 2-oxide hydrate (DEA NONOate) was prepared in 10 mM sodium hydroxide and was stored at −80 °C until further use.

### 2.2 Generation of mutant strains

For the construction of Δ*laj2*, Δ*laj3*, Δ*laj4*, and Δ*laj12* mutants, the corresponding gene was inactivated by in-frame deletion via homologous double-crossover in wild-type *S. qinglanensis*. Briefly, two homologous arms were amplified by PCR using the genomic DNA of *S. qinglanensis* as a template. These segments were used to construct a gene-knockout plasmid. The obtained plasmids were then introduced into the methylation-deficient host *E. coli* ET12567/pUZ8002 for *E. coli-Streptomyces qinglanensis* conjugation. Exconjugants were obtained after selection for apramycin resistance. After several rounds of non-selective growth, replica plating was performed, and PCR was used to screen the apramycin-sensitive colonies for the disrupted mutants.

For the overexpression of LuxR-family regulator Laj1 in *S*. *qinglanensis*, the coding region of *laj1* was amplified by PCR, and inserted into *Nde*I/*Xba*I site of pIJ8660ermE*pMCS [23]. After confirmation by DNA sequencing, the resulting integrative plasmid with *laj1* was introduced into corresponding mutants by conjugation.

### 2.3 Metabolic analysis for the *streptomyces* strains

For isotope-labeling of lajollamycin, spore suspensions of strain *Streptomyces qinglanensis* + *laj1* were used to inoculate 250-mL flasks containing 25 mL of tryptic soy broth (TSB) medium, which were incubated with shaking for 24 h at 30 °C. A 0.25 mL seed culture was then used to inoculate 100 mL flasks containing 20 mL of modified R5 medium [(g/L): K_2_SO_4_ (0.25), MgCl_2_·6H_2_O (0.25), glucose (10.0), casamino acid (0.1), yeast extract (5.0), CaCO_3_ (2.0), trace elements 2 mL/L] and these were incubated with shaking at 30 °C. l-arginine-(guanidineimino-^15^N_2_) was added at a final concentration of 3 mM at 24 h. After another 24 h, cultures were subjected to ethyl acetate extraction and concentration, and then redissolved in methanol before HPLC or HRMS analysis.

### 2.4 Fermentation, extraction, and isolation

For the production of lajollamycins, strain *Streptomyces qinglanensis* + *laj1* spores were inoculated into 750 mL of R5 medium in 2 L flasks, cultured at 180 rpm, 30°C. After 2–3 days, the whole culture was extracted twice using EtOAc. The crude extract was combined and subjected to reversed phase C18 column chromatography, eluted with 20%, 40%, 60%, 80%, and 100% MeOH in H_2_O (200 mL each). Fractions containing lajollamycins, tracked by HPLC, were fractionated on Sephadex LH-20 with MeOH elution. Fractions containing lajollamycins were further subjected to semipreparative HPLC (YMC-Triart C18, 5 μm, 10 mm ID × 250 mm) at a flow rate of 3 mL/min with 49% CH_3_CN in H_2_O. Strain Δ*laj2*-*laj1* and Δ*laj3*-*laj1* were used to produce denitrolajollamycin (**4**), denitrolajollamycin (**4**) was extracted and purified similarly as described above.

### 2.5 Protein expression and purification

The genes of *laj2*, ferredoxin reductases (FdR) and ferredoxins (Fdx) were PCR-amplified using the genomic DNA of *Streptomyces qinglanensis* as a template. The resulting segments were cloned into vector pET28a to afford expression plasmids. These plasmids were transformed into *E. coli* Transetta (DE3) cells for protein expression. To prepare starting culture, the resulting transformants were grown overnight in Luria-Bertani (LB) broth containing kanamycin (50 μg mL^−1^) at 37 °C and 200 rpm. A starting culture (750 μL) was then used to inoculate LB broth (750 mL) containing 50 μg mL^−1^ kanamycin. The culture was grown at 37 °C and 180 rpm to an optical density of 0.6 at 600 nm, and then cooled to 18 °C. Isopropyl β-d-1-thiogalactopyranoside (IPTG, final concentration 0.1 mM), ammonium ferrous sulfate hexahydrate (final concentration 1 mM) and 5-aminolevulinic acid (final concentration 1 mM) were added to induce overproduction of the protein. After 18 h of further incubation, the cells were harvested and resuspended in lysis buffer (300 mM NaCl, 50 mM Tris-HCl, 10 mM imidazole, pH 8.0) and applied to sonication. Cell debris was removed by centrifugation at 15,000 rpm for 40 min, and the supernatant was mixed with 1 mL of Ni-NTA agarose for 1 h at 4 °C. After being washed with washing buffer (300 mM NaCl, 50 mM Tris-HCl, 50 mM imidazole, pH 8.0), protein was eluted with elution buffer (300 mM NaCl, 50 mM Tris-HCl, 300 mM imidazole, pH 8.0). The target proteins were confirmed by SDS-PAGE analysis, and then dialyzed into the storage buffer (25 mM HEPES, 100 mM NaCl, 2 mM DTT, 10% glycerol, pH 7.4). The target proteins were then concentrated with size-exclusion filters (3 kDa for Fdxs, 10 kDa for P450 and FdRs) (Millipore) and stored at −80 °C for further use. Protein concentration was determined by using the Bradford protein assay (Bio Basic Inc). The concentrations of ferredoxins and ferredoxin reductases were determined by previously reported extinction coefficients [24].

### 2.6 Determination of dissociation constant

A solution of Laj2 (1800 μL, 14 μM) in HEPES buffer (25 mM, pH 7.4) was divided equally between two cuvettes. Denitrolajollamycin (**4**) was added to the sample cuvette (0.5 μL, 20 mM solution in DMSO) and an equal volume of DMSO was added to the reference. Difference spectra was measured by Shimadzu UV-2600 spectrophotometer. The difference in absorbance of each spectrum at the *λ*_max_ (389 nm) and the λ_min_ (424 nm) was calculated and plotted against the concentration of the substrate. The data were fitted to a one site binding model [25] using Origin 8.5.

### 2.7 In vitro biochemical assays

For the in vitro assay of Laj2, the reaction mixture (200 μL) contained 9 μM P450, 30 μM Fdx, 30 μM FdR, 0.25 mM substrate, 0.25 mM Diethylamine NONOate, and 2 mM NADPH in reaction buffer (25 mM HEPES, pH 7.4). The mixture was incubated at 30°C for 30 min, and quenched by vortex mixing with equal volume of MeOH. After centrifugation, the supernatant was subjected to HPLC or LC-MS analysis.

For the in vitro assay of Laj3, the reaction mixture (50 μL) contained 5 μM Laj3, 0.5 mM N^G^-hydroxyl-l-arginine, 40 mM H_2_O_2_ in 25 mM HEPES buffer (pH 7.4). The reaction mixture was incubated for 10 min at 30 °C and quenched with 200 units of catalase, followed by nitrite detection using Griess reagent. Heat-inactivated Laj3 was used as a negative control.

## 3 Results and discussion

### 3.1 Genome mining using NOS

**Fig. 2:**
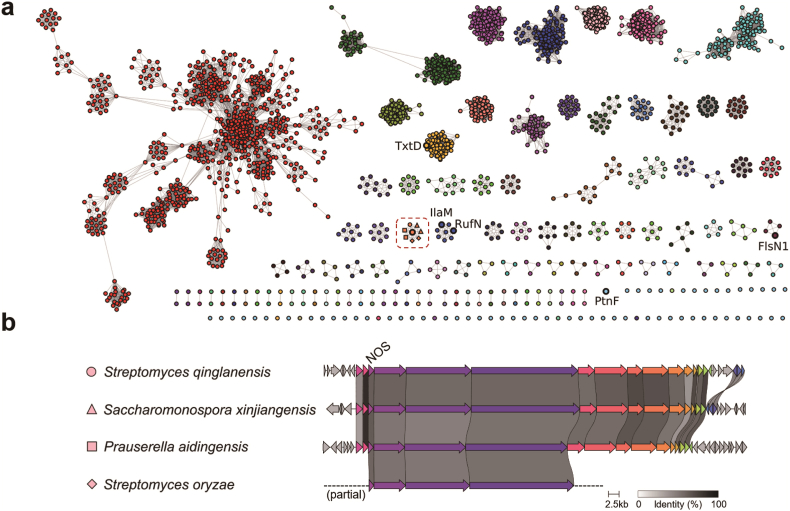
A nitric oxide synthase-genome mining strategy for targeted isolation of bacterial NO-utilizing metabolic pathways. (a) Sequence similarity network (SSN) analysis of bacterial NOSs. Note: NOSs present in the BGCs of known natural products are displayed next to the corresponding nodes. The cluster of NOSs prioritized for further investigation is boxed within red dash line. (b) The NOS-containing biosynthetic gene clusters distributed in several actinobacterial genomes are prioritized for further product analysis.

In our pursuit of identifying natural products reliant on nitric oxide (NO) as a metabolic precursor, we initiated the construction of a sequence similarity network (SSN) anchored by the NOS PtnF found within the 8-azaguanine biosynthetic gene cluster (BGC) (Fig. 2a). Homologous proteins of PtnF with sequence identity >45% were collected and used for this analysis. Notably, the SSN result included NOS genes from the BGCs responsible for thaxtomins (TxtD), rufomycins (RufN/IlaM) and pyrazolofluostatins (FlsN1). Further analysis of our SSN results using the Genome Neighborhood Tool (GNT) also revealed a cluster of NOS proteins originating from a conserved BGCs distributed across the genomes of many actinobacteria, including species from *Streptomyces*, *Saccharomonospora*, and *Prauserella* (Fig. 2b). These BGCs harbor several putative modular type I PKS and PKS/NRPS hybrid enzymes. The co-occurrence of the NOS gene alongside a P450 gene within these BGCs indicates the potential for encoding novel nitrated products. These BGCs were thus prioritized for further metabolic analysis.

### 3.2 Identification of lajollamycins as the products from the BGC

**Fig. 3:**
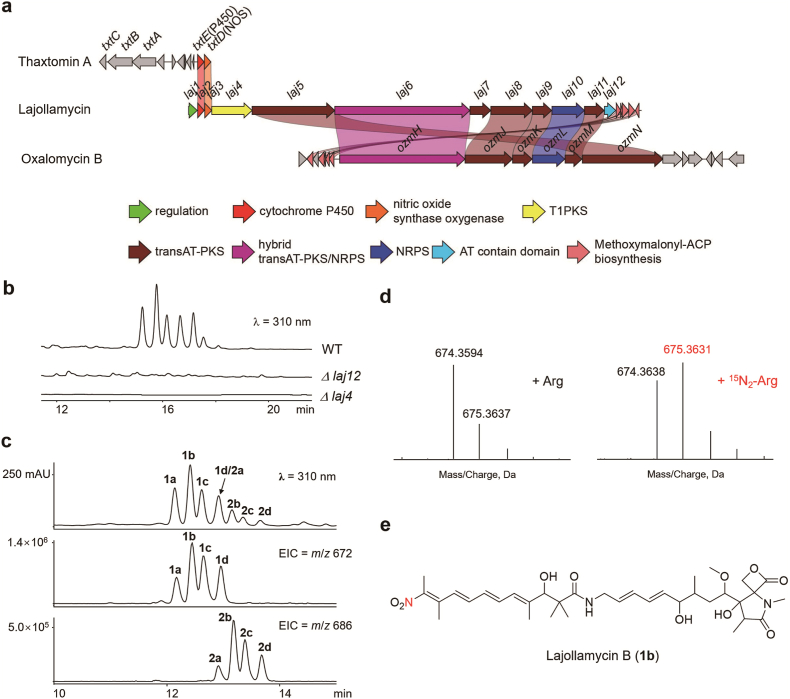
Identification of lajollamycins as the products of the NOS-containing biosynthetic gene cluster from *Streptomyces qinglanensis* CGMCC 4.6825. (a) Comparison of the *laj* BGC with the BGCs of thaxtomin A and oxazolomycin B. (b) Metabolic profiling of *Streptomyces qinglanensis* wild type (WT) strain and its mutants by HPLC analysis. (c) LC-MS analysis of the potential products encoded by the *laj* BGC. (d) LC-HR-MS analysis of the isotopic pattern of compound **1b** after the producer strain was fed with l-Arg or ^15^N_2_-l-Arg. (e) Identification of **1b** as lajollamycin B.

To identify the product(s) encoded by these NOS-containing BGCs, we focused on a BGC (∼83.4 kb) from the strain *Streptomyces qinglanensis* CGMCC 4.6825. Upon comparison with known BGCs recorded in the MIBiG database, this putative BGC (named here as the *laj* BGC) revealed similarity to the oxazolomycin BGC (*ozm* BGC) (Fig. 3a and Table 1). However, the *ozm* BGC lacks a NOS and a P450 gene [26]. We next set out to determine the product encoded by the *laj* BGC. Within this BGC, Laj4 encodes a type I PKS, while Laj12 is a putative acyltransferase (AT). We speculated that these enzymes likely play a role in the backbone assembly of the putative product. To facilitate the identification of product from *laj* BGC, we constructed the gene knock-out mutants for *laj4* and *laj12*, and subjected the resulting strains *Δlaj4* and *Δlaj12* to metabolic profiling. Compared with the parental strain, we found that both mutants abolished the production of a series of compounds displaying similar UV-visible spectra (Fig. 3b). Further LC-MS analysis of the target mixture suggested the presence of at least eight compounds, denoted as compounds **1a**-**1d** with a mass value of *m*/*z* 672 ([M − H]^-^), and **2a**-**2d** with a mass value of *m*/*z* 686 ([M − H]^-^) (Fig. 3c).

**Table 1:** Gene organization and annotation of the *laj* BGC from strain *Streptomyces qinglanensis* CGMCC 4.6825 (GenBank accession: NZ_FOGO01000010: 38,930.122,339).

Protein	Size (amino acid)	Identified/Proposed function	Homolog	Identity (%)
Laj1	532	LuxR family transcriptional regulator		
Laj2	405	cytochrome P450	TxtE	29 %
Laj3	438	nitric oxide synthase	TxtD	63 %
Laj4	2486	type I PKS	AvmA (QSV12655)	52 %
Laj5	5112	*trans*-AT PKS	OzmN	44 %
Laj6	8342	hybrid NRPS/*trans*-PKS	OzmH	49 %
Laj7	1289	*trans*-AT PKS		
Laj8	2559	*trans*-AT PKS	OzmJ	40 %
Laj9	1187	*trans*-AT PKS	OzmK	56 %
Laj10	2004	NRPS	OzmL	49 %
Laj11	1224	acyltransferase	OzmM	50 %
Laj12	697	acyltransferase		
Laj13	278	3-Hydroxyacyl-CoA-dehydrogenase	OzmG	60 %
Laj14	101	ACP	OzmE	55 %
Laj15	381	acyl-dehydrogenase	OzmD	62 %
Laj16	366	glyceryltransferase/phosphatase	OzmB	66 %
Laj17	222	O-methyltransferase	OzmF	61 %

To ascertain whether these metabolites originate from the *laj* BGC and incorporate NO as a structural subunit, we conducted a feeding experiment using l-arginine-(guanidineimino-^15^N_2_) with the wild type *S*. *qinglanensis* strain. Subsequent analysis of the isotopic pattern of these products via LC-HR-MS revealed an enrichment of +1 Da isotopic peak, supporting the hypothesis that these products indeed carry a structural moiety derived from NO (Fig. 3d). This HR-ESI-MS result (*m*/*z* 674.3594, [M + H]^+^) also suggested a molecular formula for **1b** as C_35_H_51_N_3_O_10_. We next proceeded to isolate these compounds and subjected the major component (**1b**) to detailed structural elucidation. Through a comprehensive analysis of its NMR spectra and the comparison with existing literature [[27], [28], [29]], We identified **1b** as the 10′*E* isomer of the known compound lajollamycin B (Fig. 3e and Figure S1). The 8′*E* and 10′*E* geometries were established by ROESY correlations from 9′H to 11′CH_3_ and from 10′CH_3_ to 8′H. The 4′*E* and 6′*E* configurations were assigned on the basis of the ROESY correlations between 3′H and 5′H, 4′CH_3_ and 6′H, 5′H and 7′H (Figure S1). We noticed that **1a**-**1d** can convert to each other at room temperature, indicating that they are all isomers of lajollamycin B (Figure S2). Compounds **2a-2d**, which can also convert to each other, were assigned as lajollamycin C isomers based on their values (*m*/*z* 686, [M − H]^-^ ion) [28]. The structural variance between lajollamycin B and C likely stems from the different amino acids (l-serine for **1a**-**1d** or l-threonine for **2a**-**2d**) incorporated by the NRPS Laj10 (Table 1).

Lajollamycin was initially isolated from marine *Streptomyces nodosus* [27], and its analogs were later also identified as products from *Streptomyces* strain SMC72 [28]. However, the BGC responsible for lajollamycin biosynthesis remained unidentified. On the other hand, while metabolic analysis of strain *S*. *qinglanensis* CGMCC 4.6825 has been previously conducted, leading to the characterization of several new compounds, this strain is not recognized as a lajollamycin producer [30]. Notably, we observed that lajollamycins demonstrated instability under light exposure, yielding decomposition products such as (3*E*,5*E*,7*E*)-3-methyldeca-3,5,7-triene-2,9-dione (**3**) (HR-ESI-MS: *m*/*z* 179.1063, [M + H]^+^) (Figure S3), which was previously reported as a product from *S*. *qinglanensis* CGMCC 4.6825 [30]. Taken together, these results supported that the BGC identified via NOS-guided genome mining is responsible for lajollamycin biosynthesis.

### 3.3 Identification of denitrolajollamycin as a pathway intermediate

**Fig. 4:**
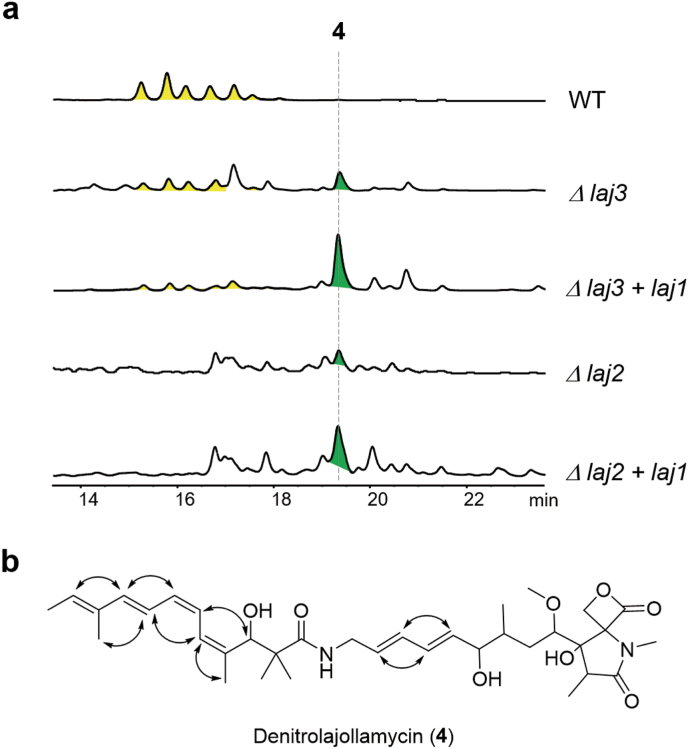
Identification of denitrolajollamycin (**4**) as a product from the gene knockout mutants of *laj2* and *laj3*. (a) Metabolic analysis of *Streptomyces qinglanensis* wild type (WT) strain and its mutants by HPLC analysis. Note: lajollamycins are indicated in yellow, denitrolajollamycin is shown in green. (b) The structure of denitrolajollamycin (**4**). Arrows indicate the key ROESY correlations observed in NMR analysis.

Considering that nitration of a linear substrate is rare in natural product biosynthesis, we proceeded to investigate the unique olefin nitration process within the lajollamycin pathway. Individual deletions of the NOS gene *laj3*, and the potential nitrating P450 enzyme *laj2*, were executed in strain *S*. *qinglanensis* CGMCC 4.6825, followed by metabolic profiling of the resulting strains. We found that the mutant *Δlaj3* can still produce small amounts of lajollamycins, whereas strain *Δlaj2* completely abolished lajollamycin production (Fig. 4a). Moreover, both mutants *Δlaj2* and *Δlaj3* accumulated a compound (**4**) displaying a mass value of 627.3684, which is consistent with the [M − H]^-^ ion of a denitro-derivative of **1a**-**1d**.

To facilitate the isolation of **4** for structural characterization, we first overexpressed the potential pathway activator gene *laj1*, a member of the LuxR family transcriptional regulators, in mutants *Δlaj2* and *Δlaj3*. We then purified **4** from a scale-up culture of strain *Δlaj3* + *laj1* (Fig. 4a). Subsequent NMR analysis confirmed the identity of **4** as denitrolajollamycin B, which lacks a nitro group compared with the structure of lajollamycin B. The configuration of tetranene moiety of **4** was determined based on COSY, HSQC, HMBC, ROESY (Figure S4). It is worth noting that the configuration of the tetraene moiety of **4** differs from that of **1b**, which may arise from a non-enzymatic conversion. Collectively, these results supported the involvement of Laj2 and Laj3 in the nitration reaction within lajollamycin biosynthesis. The residual production of lajollamycins observed in strain *Δlaj3* likely arise from genetic complementation by other NO-generating pathway(s) from the host.

### 3.4 In vitro biochemical assays for Laj3 and Laj2

**Fig. 5:**
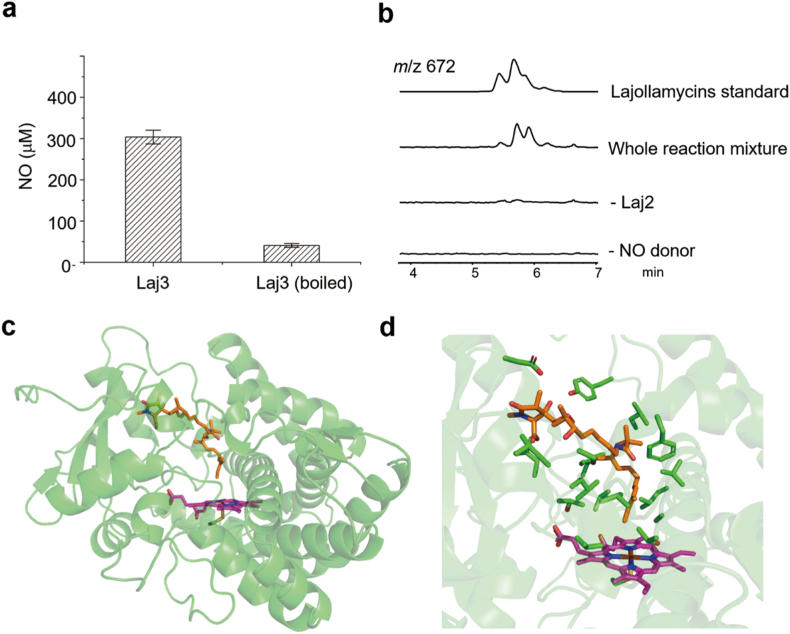
In vitro biochemical studies of Laj3 and Laj2. (a) In vitro study of the NOS Laj3. (b) In vitro study of the nitrating P450 enzyme Laj2. Note: the whole reaction mixture contains Laj2, **4**, NO donor, NADPH, spinach Fdx and FdR. Assays lacking Laj2 or NO donor were used as a control. (c) In silico study of the binding mode of Laj2 with **4** by molecular docking. Note: an AlphaFold-predicted structure of Laj2 is used for this study. The heme cofactor is shown in purple and compound **4** is shown in orange. (d) Interaction of **4** with the active site residues of Laj2.

We next interrogated the role of Laj3 and Laj2 in vitro. His_6_-tagged Laj3 and Laj2 were expressed and isolated from the *Escherichia coli* system. In silico analysis revealed that Laj3 shares 63% sequence identity with TxtD, a well-characterized NOS from the biosynthetic pathway of thaxtomins. To validate the role of Laj3 as a bacterial NOS, we assessed its activity in converting *N*^G^-hydroxyl-l-arginine to NO in the presence of hydrogen peroxide (H_2_O_2_), which is a widely employed in vitro assay for NOS characterization [15]. Upon quenching the reactions with catalase, we detected a considerable amount of nitrite using the Griess reagent (Fig. 5a), whereas only negligible amount of nitrite was detected from a control assay in which inactivated Laj3 was used. These results demonstrated that Laj3 is a canonical NOS.

The accumulation of **4** in mutants *Δlaj2* and *Δlaj3* suggested that nitration likely occurs as a post-assembly line modification. While P450-catalyzed aromatic nitration was reported previously, nitration on a linear substrate by P450 enzyme has not been described in natural product biosynthesis. In silico analysis of Laj2 showed that it shares 46.5 % and 47.1 % sequence identity with TamI (PDB: 6XA2) and PikC (PDB: 2WHW), respectively. Both TamI and PikC are P450 monooxygenases catalyzing post-assembly line hydroxylation in the biosynthesis of polyketide natural products [31,32]. Moreover, we found that the AlphaFold-predicted structural model of Laj2 well superposed with the crystal structures of TamI or PikC (Figure S5). We then titrated Laj2 with isolated **4**, and observed the characteristic type I binding spectra, with a dissociation constant estimated to be ∼60 μM (Figure S6).

We next incubated Laj2 with **4**, the NO donor NONOate, NADPH, spinach ferredoxin (Fdx) and ferredoxin reductase (FdR), followed by LC-MS analysis of the reaction mixture. We observed that lajollamycin B isomers were generated in the above mixture, and their production were dependent on both Laj2 and NONOate, demonstrating the role of Laj2 in catalyzing the nitration of **4** using NO and O_2_ (Fig. 5b and Figure S7). However, the conversion rate of this reaction is relatively low (∼3%). We speculated that the spinach Fdx and FdR used in this vitro assay might not be compatible with Laj2. In an attempt to enhance the conversion rate, we evaluated three pairs of commonly-used P450 redox partners, along with three putative FdRs and five Fdxs from *S. qinglanensis* (Table S3), for their potential support of multiple turnovers in this nitration reaction. Unfortunately, none of these components increased the conversion rate, suggesting utilization of alternative pathways by *S. qinglanensis* to reduce the heme iron of Laj2. It should be mentioned that we cannot exclude the possibility that Laj2 might catalyze on-line nitration of a carrier protein-tethered pathway intermediate, instead of **4**. Nevertheless, this discovery marks Laj2 as the first identified P450 enzyme capable of catalyzing nitration on a linear substrate in natural product biosynthesis.

To further study the binding mode of **4** with Laj2, we performed molecular docking analysis using the AlphaFold-predicted structural model of Laj2 (Fig. 5c). The result showed that **4** can be well docked into the putative active site of Laj2, where a significant number of hydrophobic residues were found (Fig. 5d). These residues could potentially interact with the tetraene moiety of **4**.

### 3.5 Proposed biosynthetic pathway for lajollamycins

**Fig. 6:**
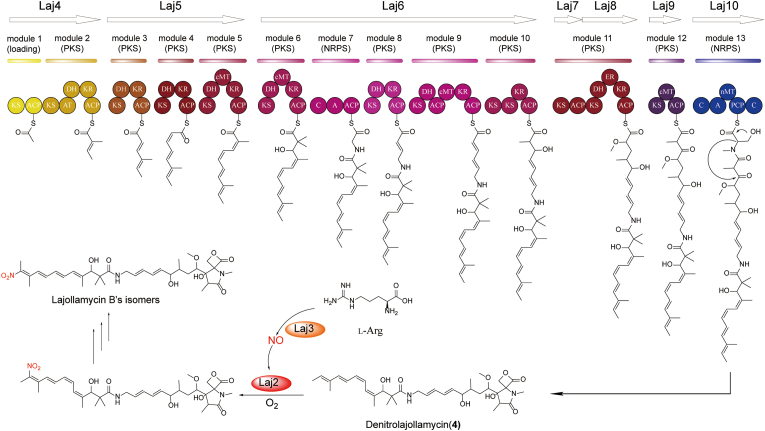
A proposed biosynthetic pathway for lajollamycins.

Based on the results from our above genetic and biochemical studies, as well as the current knowledge about the biosynthetic pathway of oxazolomycin [26,33], we propose a biosynthetic pathway for lajollamycins (Fig. 6). The biosynthesis of lajollamycin is initiated by the PKS Laj4, which has a domain organization of KS-ACP-KS-AT-DH-KR-ACP, with its AT domain predicted to exhibit specificity towards methylmalonyl-CoA. Following this initiation, Laj4's product undergoes sequential transfer to Laj5-Laj10, ultimately resulting in the release of intermediate **4** catalyzed by Laj10. Notably, the *laj* BGC encodes two distinct acyltransferases, Laj11 and Laj12. Laj11, sharing sequence similarity with OzmM from the oxazolomycin pathway, is presumed to facilitate the loading of extender units (malonyl-CoA) for all the AT-less PKS modules, while Laj12 is envisioned to load the methoxymalonyl-ACP extender unit specifically for PKS Laj8. The liberated intermediate **4**, subsequently subjected to Laj2-catalyzed reactions, undergoes nitration, leading to the generation of a nitrated product. This product undergoes further transformations, giving rise to the diverse spectrum of lajollamycin isomers observed.

## 4 Conclusion

Nitro-containing compounds display diversified chemical structures and significant biological activities. Consequently, natural and engineered nitrating enzymes have emerged as invaluable biocatalysts in synthetic biology and biocatalysis, garnering substantial attention. In this study, by using a nitric oxide synthase-guided genome mining approach, we identified the biosynthetic gene cluster responsible for the production of the nitro-containing antibiotic lajollamycins. Delving deeper into its enzymatic foundation unraveled insights into the creation of its distinctive nitro-tetraene structure, uncovering the first cytochrome P450 enzyme capable of catalyzing olefin nitration within natural product biosynthesis. Our results not only present an efficient strategy for targeted isolation of nitrated natural products, but should also facilitate further development of promising nitrating biocatalysts.

## CRediT authorship contribution statement

**Hu Li:** Data curation, Formal analysis, Investigation, Visualization, Writing – original draft. **Wei Li:** Investigation. **Kaihui Song:** Investigation. **Yu Liu:** Investigation. **Guiyun Zhao:** Investigation. **Yi-Ling Du:** Conceptualization, Funding acquisition, Methodology, Project administration, Resources, Supervision, Validation, Writing – original draft, Writing – review & editing.

## Declaration of competing interest

The authors declare that they have no known competing financial interests or personal relationships that could have appeared to influence the work reported in this paper.
