# Supplementary material for: Nitric oxide synthase-guided genome mining identifies a cytochrome P450 enzyme for olefin nitration in bacterial specialized metabolism
Source: Synth Syst Biotechnol. 2024 Jan 17;9(1):127–33. doi: 10.1016/j.synbio.2024.01.005 (PMC10831120; doi:10.1016/j.synbio.2024.01.005)
Supplement: Multimedia component 1 [file mmc1.pdf]

Supporting Information

Nitric oxide synthase-guided genome mining identifies a cytochrome P450 enzyme for olefin nitration in bacterial specialized metabolism

Hu Li [a], Wei Li [b], Kaihui Song [b], Yu Liu [c], Guiyun Zhao [b], Yi-Ling Du [a,b] *

[a] Polytechnic Institute, Zhejiang University, Hangzhou, 310022, China.
[b] Department of Microbiology, Zhejiang University School of Medicine, Hangzhou, 310058, China
[a] College of Life Sciences, Zhejiang University, 310058, Hangzhou, China.
*E-mail address: yldu@zju.edu.cn (Y.-L. Du)

Table S1 Primers used in this study

Primers	Sequence(5'→3')	Description
P258-LuxR-F	ACCAAAGGAGGCGGACATatggcgcgatccttccgccggg	Primers for overexpression of *laj1*
P258-LuxR-R	CCGCAAGCTTAGTCTAGAaagggcgtctcgaacctatggtcctg	
P450-KO-LAF	tacgaagagcttttataaggtaccgcaccggccagttcgtccagtc	Primers for deletion of *laj2*
P450-KO-LAR	ttctcgggtgtgccggaccggcctgagcacactgccgatc	
P450-KO-RAF	ggcagtgtgctcaggccggtccggcacacccgagaagtcc	
P450-KO-RAR	gtggtggtggtggtggtgaagcttaacctgactccgtcgtgccagg	
NOS-KO-LAF	gatatccacctcgagcacccgggcgaactcggtgaacatg	Primers for deletion of *laj3*
NOS-KO-LAR	gcctcttccacagcgaggctcggccaccctcgtcttccac	
NOS-KO-RAF	aagacgagggtggccgagcctcgctgtggaagaggccgac	
NOS-KO-RAR	gtggtggtggtggtggtgaagcttggagctggccaggatcggtacc	
AT-KO-LAF	tacgaagagcttttataaggtaccgttcctccaccctgctgagttc	Primers for deletion of *laj12*
AT -KO-LAR	cgttctcccggaggtttcgctcatgaccggactggatgtg	
AT -KO-RAF	tccagtccggtcatgagcgaaacctccgggagaacggaac	
AT -KO-RAR	gtggtggtggtggtggtgaagcttgagcactactggcaggtggtct	
C3 -KO-LAF	tacgaagagcttttataaggtacccagcccggtgccgtattccagc	Primers for deletion of *laj4*
C3-KO-LAR	cagttctgggacaacctgcggatggctgctcggccagttg	
C3-KO-RAF	tggccgagcagccatccgcaggttgtcccagaactgcgtc	
C3-KO-RAR	gtggtggtggtggtggtgaagcttcgacatcaagctgaacaccgtc	
P450-XhoI-F	agcagcCTCGAGtcagtgcagccggatcggcagt	Primers for protein expression of Laj2
P450-NdeI-R	agcagcCATatgacgaccttgaccgggcccg	
NOS-XhoI-F	agcagcCTCGAGtcatccggtgatccctcctggg	Primers for protein expression of Laj3

NOS-NdeI-R	agcagcCATATGctcagtgaaagtctctttcggc	
Fdx1-F	gtgccgcgcggcagccatATGACCGATCGCTGGCATGTCG	Primers for protein expression of putative ferredoxins 1
Fdx1-R	gtggtggtggtggtggtgctcgagTCAGTCCTCCGGCGGGAAGACC	
Fdx2-F	gtgccgcgcggcagccatgtgacgggggagcccgcggcag	Primers for protein expression of putative ferredoxins 2
Fdx2-R	gtggtggtggtggtggtgctcgagctacggggcgacgggtgcgccg	
Fdx3-F	gtgccgcgcggcagccatatgctccgcgtgaccgcgaacc	Primers for protein expression of putative ferredoxins 3
Fdx3-R	gtggtggtggtggtggtgctcgagtcactcctcggtgacgtggatg	
Fdx4-F	gtgccgcgcggcagccatATGAAACTCCTGCTGGACTCCA	Primers for protein expression of putative ferredoxins 4
Fdx4-R	gtggtggtggtggtggtgctcgagTCACTTCTCCAGCCTGAGCGCG	
Fdx5-F	gtgccgcgcggcagccatGTGACTTACGTCATCGCGGAGC	Primers for protein expression of putative ferredoxins 5
Fdx5-R	gtggtggtggtggtggtgctcgagTCACTCGTCGTGCTCCTGCGGC	
FdR1-R	gtggtggtggtggtggtgctcgagTCAGGCCGGACCGGCGGCGCCC	Primers for protein expression of putative ferredoxin reductases 1
FdR1-F	gtgccgcgcggcagccatGTGCCGCGGAACTCGCCGCGTG	
FdR2-R	gtggtggtggtggtggtgctcgagTCATCGGCGTCTCCTCCTGCCC	Primers for protein expression of putative ferredoxin reductases 2
FdR2-F	gtgccgcgcggcagccatATGACCTCGCCCTCCCCGGACG	
FdR3-R	gtggtggtggtggtggtgctcgagTCAGCGGGGGAGCGGGAGGCGG	Primers for protein expression of putative ferredoxin reductases 3
FdR3-F	gtgccgcgcggcagccatATGACCTACGCCATCAGCCAGA	

Table S2. Strains used in this study

Strains	Description	Source
Streptomyces qinglanensis CGMCC 4.6825	Wild-type Lajollamycin producer	China General Microbiological Culture Collection Center, (CGMCC)
Δ*laj2*	CGMCC 4.6825(Δ*laj2*)	This study
Δ*laj3*	CGMCC 4.6825(Δ*laj3*)	This study
Δ*laj4*	CGMCC 4.6825(Δ*laj4*)	This study
Δ*laj12*	CGMCC 4.6825(Δ*laj12*)	This study
WT-*laj1*	CGMCC 4.6825+ *laj1*	This study
Δ*laj2+laj1*	CGMCC 4.6825(Δ*laj2*)+ *laj1*	This study
Δ*laj3+laj1*	CGMCC 4.6825(Δ*laj3*)+ *laj1*	This study
E. coli DH5α	General cloning host	Laboratory stock
E. coli ET12567/ pUZ8002	Methylation-deficient strain used for conjugation	Laboratory stock
E. coli Transetta	Host for protein heterologous expression	Laboratory stock

Table S3. Putative ferredoxins (Fdx) and ferredoxin reductases (FdR) from *S. qinglanensis* used in this study.

FdR	Accession
4.6825-FdR1	WP_074998610.1
4.6825-FdR2	SES13380.1
4.6825-FdR3	WP_075001892.1
Fdx	
4.6825-Fdx1	WP_074998542.1
4.6825-Fdx2	WP_074999506.1
4.6825-Fdx3	WP_074998165.1
4.6825-Fdx4	WP_075000137.1
4.6825-Fdx5	WP_019354435.1

Note: Commonly-used redox partners for P450 enzymes were also tested, including seFdx/seFdR from *Synechococcus elongatus* [1], CamB (putidaredoxin)/CamA (putidaredoxin reductase) from *Pseudomonas putida* [2], anFdx/anFdR from anabaena PCC7119, and RhFRED from *Rhodococcus* [3].

Figure S1. NMR spectra of **1b**

(a)

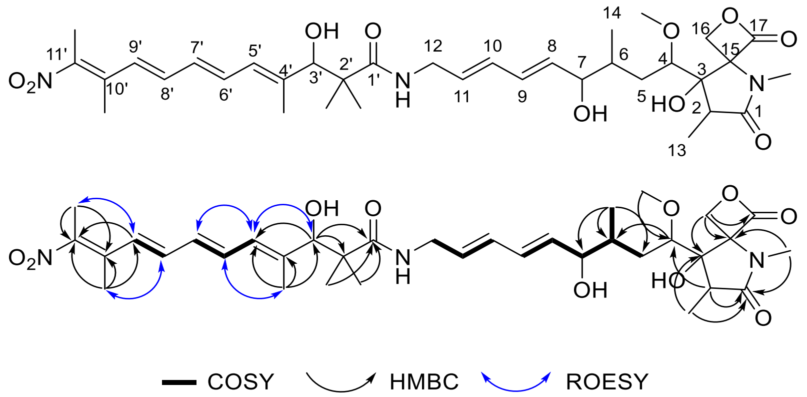

^{1}H and ^{13}C NMR data of **1b** (600 MHz, 150 MHz, $CDCl_3$)

Position	δC	δH(*J* in Hz)
1	175.0	
2	42.3	2.46(q, 7.2)
3	79.6	
4	82.4	3.58(m)
5	32.8	1.34(m), 2.04(m)
6	37.2	1.79(m)
7	76.9	3.95(m)
8	134.1	5.68(m)
9	131.1	6.19(m)
10	131.6	6.19(m)
11	129.6	5.68(m)
12	41.2	3.91(m)
13	10.1	1.21(d, 7.1)
14	17.4	0.97(d, 6.5)
15	85.9	
16	65.6	4.72(6.1), 4.38(6.1)
17	170.1	
1'	177.8	
2'	45.0	
2'-Me	25.6	1.29(s)
2'-Me	21.5	1.10(s)
3'	83.7	4.03(s)
4'	140.9	
4'-Me	13.7	1.81(s)
5'	128.4	6.08(d, 11.0)
6'	132.2	6.62(m)
7'	132.6	6.36(m)
8'	137.0	6.72(m)
9'	128.6	6.42(d, 14.7)

10'	133.7	
10'-Me	15.1	2.05(s)
11'	145.6	
11'-Me	15.9	2.30(s)
N-Me	26.2	2.91(s)
O-Me	57.1	3.38(s)

(b)

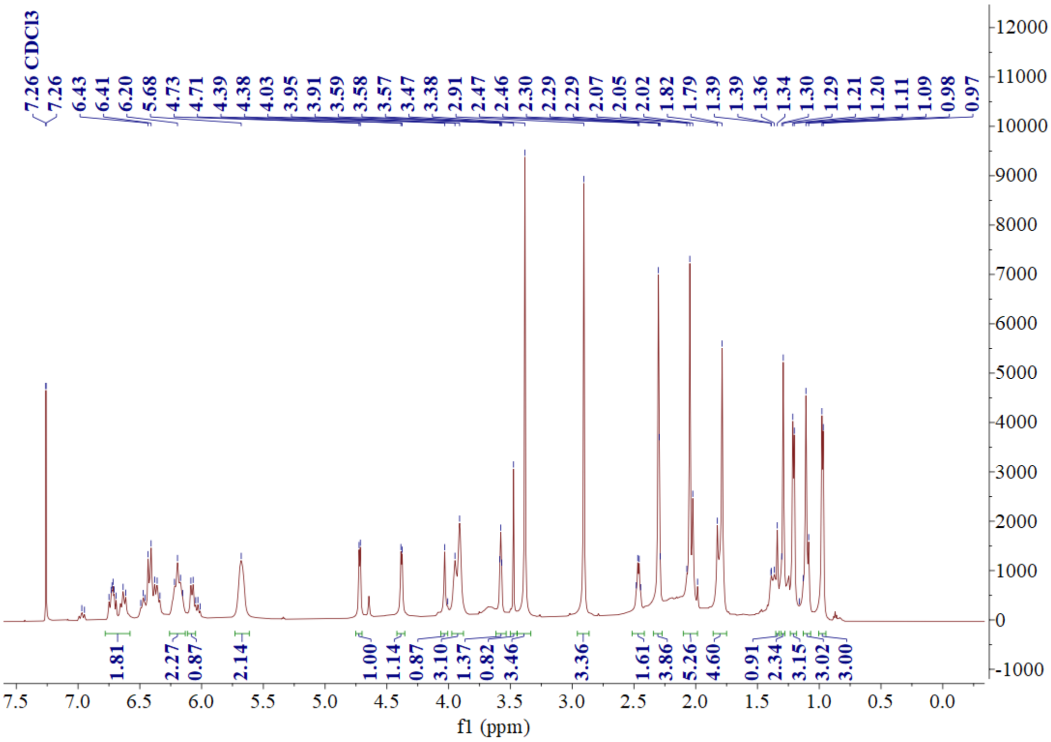

(c)

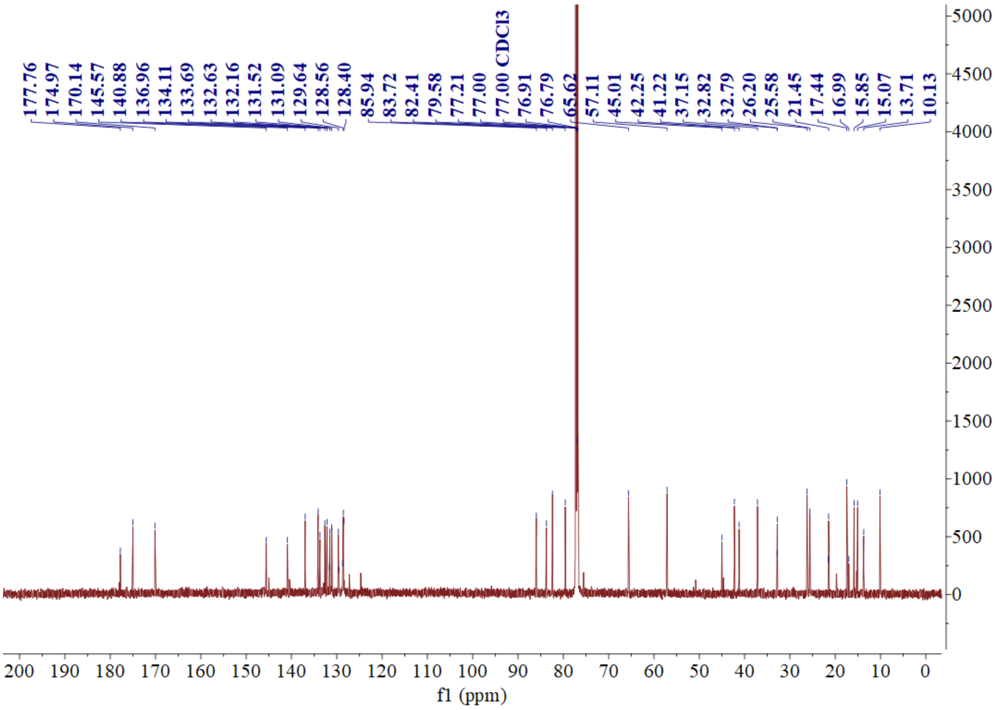

(d)

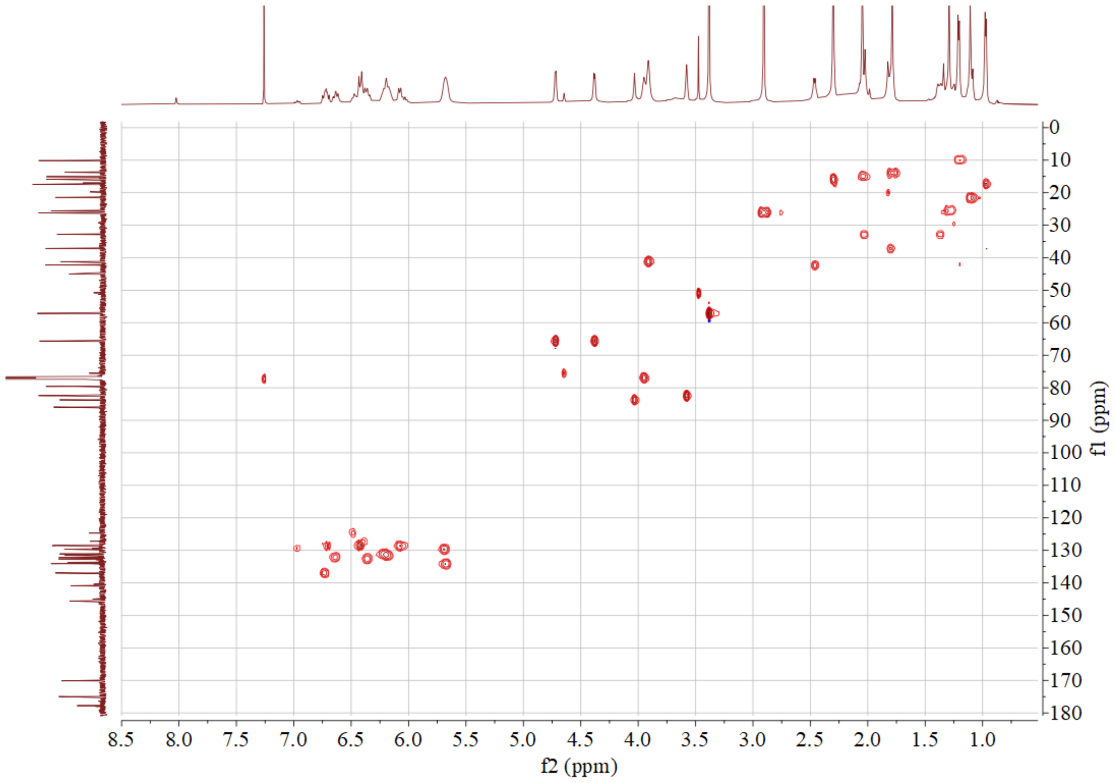

0
10
20
30
40
50
60
70
80
90
100
110
120
130
140
150
160
170
180
f1 (ppm)
8.5 8.0 7.5 7.0 6.5 6.0 5.5 5.0 4.5 4.0 3.5 3.0 2.5 2.0 1.5 1.0
f2 (ppm)

(e)

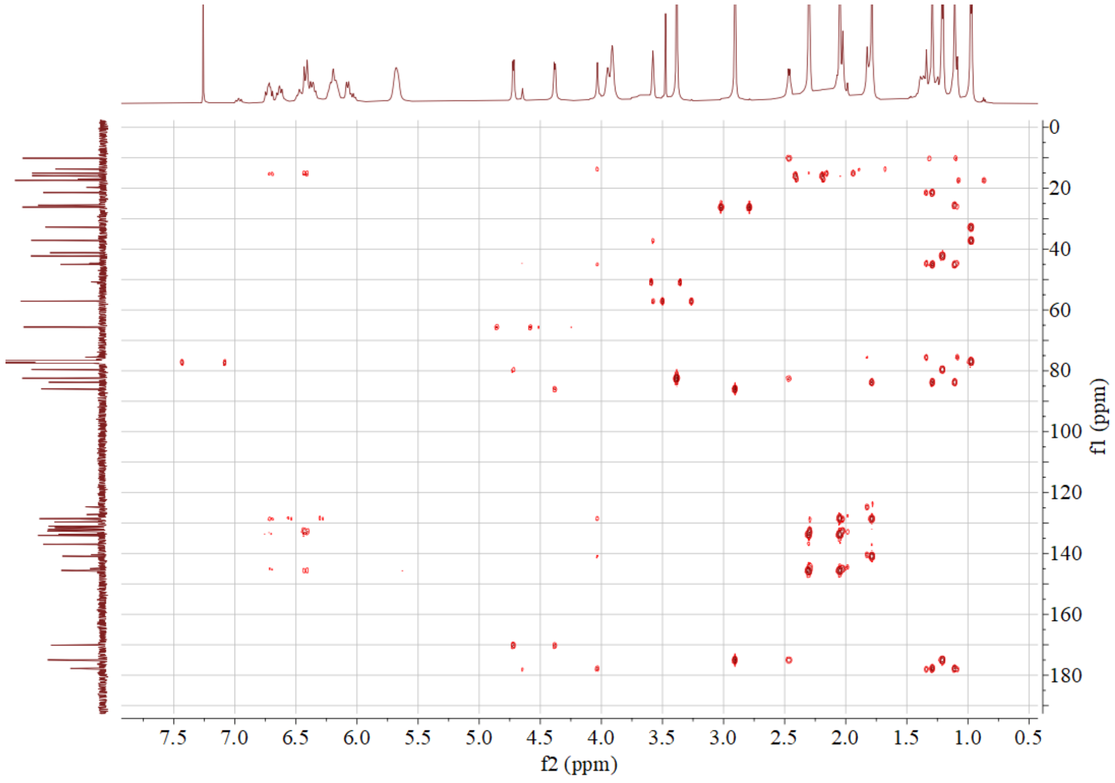

0
20
40
60
80
100
120
140
160
180
f1 (ppm)
7.5 7.0 6.5 6.0 5.5 5.0 4.5 4.0 3.5 3.0 2.5 2.0 1.5 1.0 0.5
f2 (ppm)

(f)

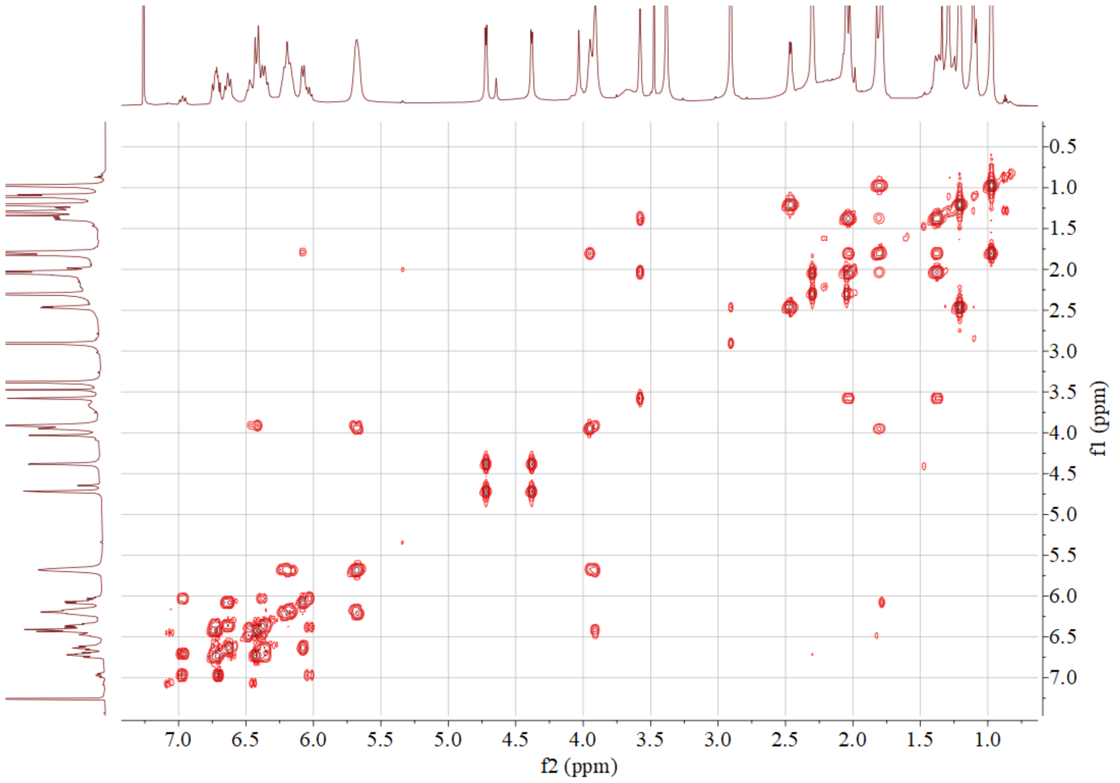

(g)

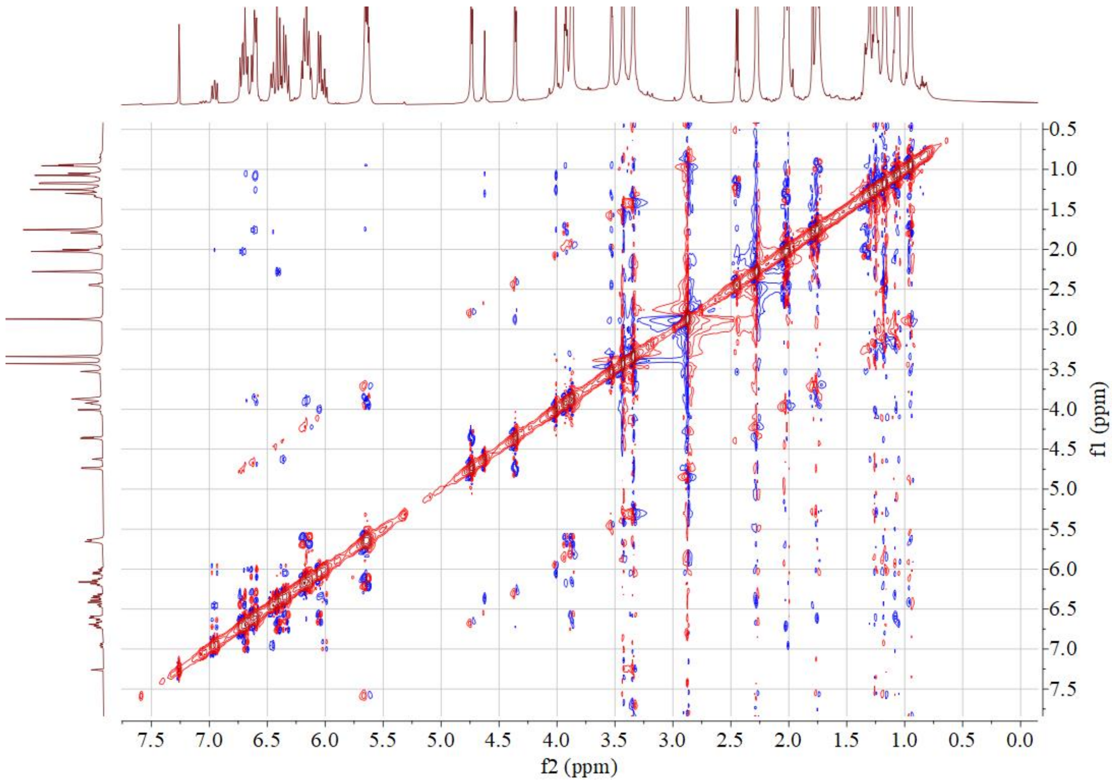

Figure S1. NMR data of lajollamycin B **(1b)**. (a) Summary of the NMR data for **1b**. (b) ^{1}H NMR spectrum of **1b**. (c) ^{13}C NMR spectrum of **1b**. (d) HSQC spectrum of **1b**. (e) HMBC spectrum of **1b**. (f) ^{1}H-^{1}H-COSY spectrum of **1b**. (g) ROESY spectrum of **1b**.

Figure S2. Spontaneous isomerization of isolated lajollamycins

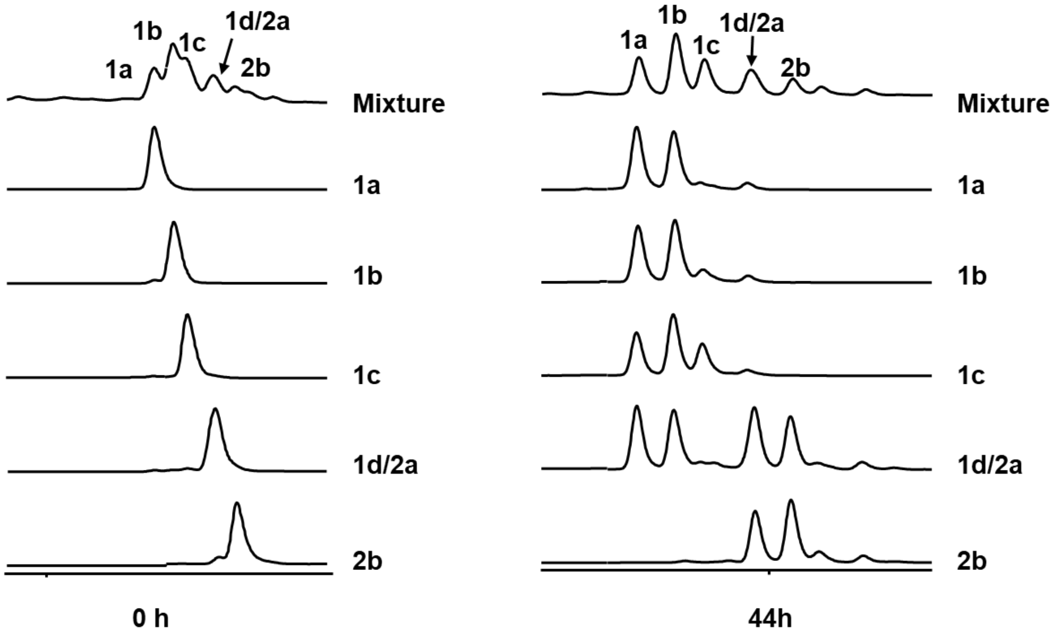

Figure S2. Detection of the spontaneous isomerization of isolated lajollamycins by HPLC (detection wavelength at λ = 310 nm). The purified lajollamycins isomers are relatively stable when stored at -20 °C in dark, and can convert to each other at room temperature (left: isolated compounds at 0 h, right: compounds after 44 h).

Figure S3. NMR data of **3**

(a)

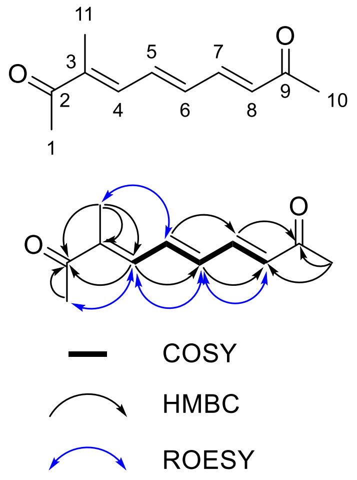

^{1}H and ^{13}C NMR data of **3** (600 MHz, 150 MHz, $CDCl_3$)

Position	δ_C	δ_H (*J* in Hz)
1	25.93	2.38(s)
2	199.54	
3	140.05	
4	137.17	7.08(d, 11.3)
5	136.26	6.96(dd, 11.4, 14.7)
6	136.79	6.66(dd, 11.3, 14.7)
7	141.93	7.23(dd, 11.2, 15.6)
8	132.74	6.28(d, 15.6)
9	198.33	
10	27.36	2.32(s)
11	12.09	1.96(s)

(b)

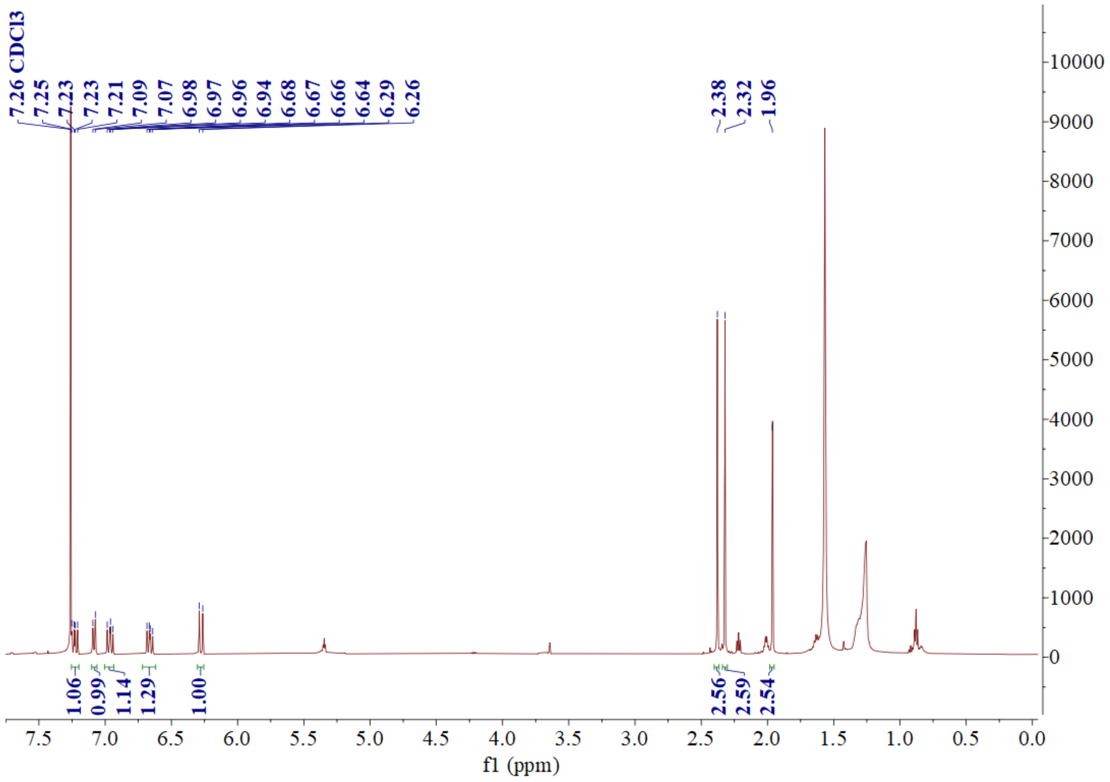
7.26 CDCl3
7.25
7.23
7.23
7.21
7.09
7.07
6.98
6.97
6.96
6.94
6.68
6.67
6.66
6.64
6.29
6.26
2.38
2.32
1.96
1.06
0.99
1.14
1.29
1.00
2.56
2.59
2.54
f1 (ppm)

(c)

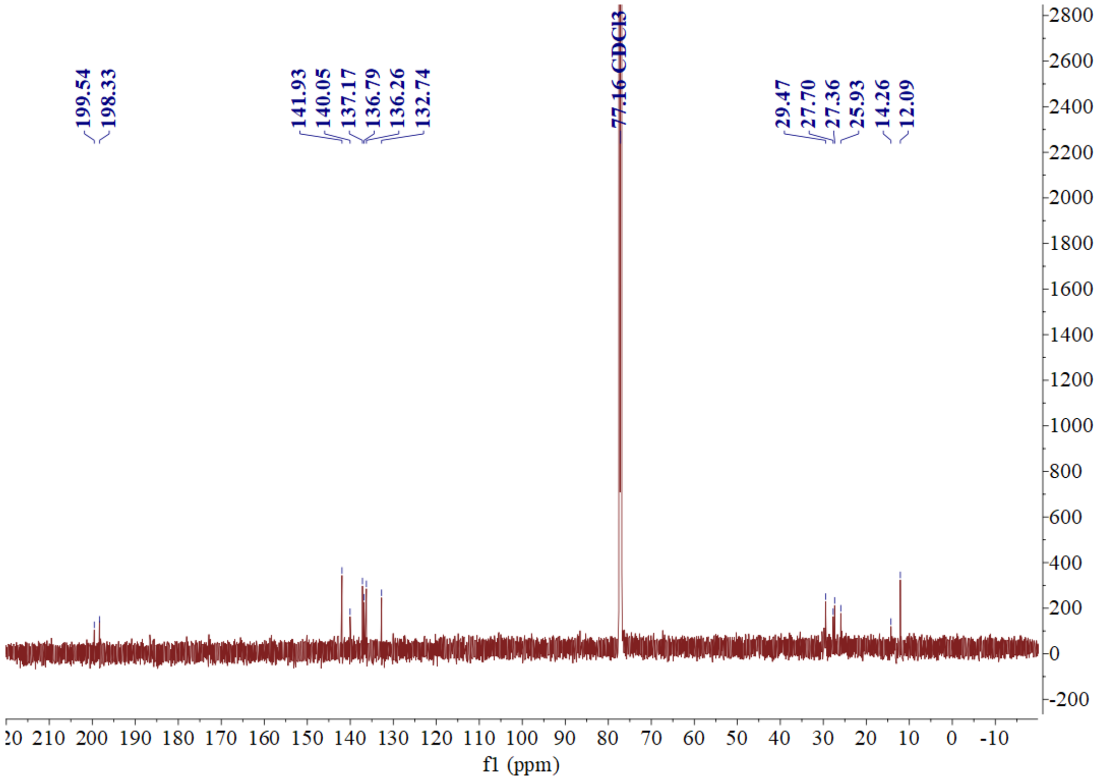
199.54
198.33
141.93
140.05
137.17
136.79
136.26
132.74
77.16 CDCl3
29.47
27.70
27.36
25.93
14.26
12.09
f1 (ppm)

(d)

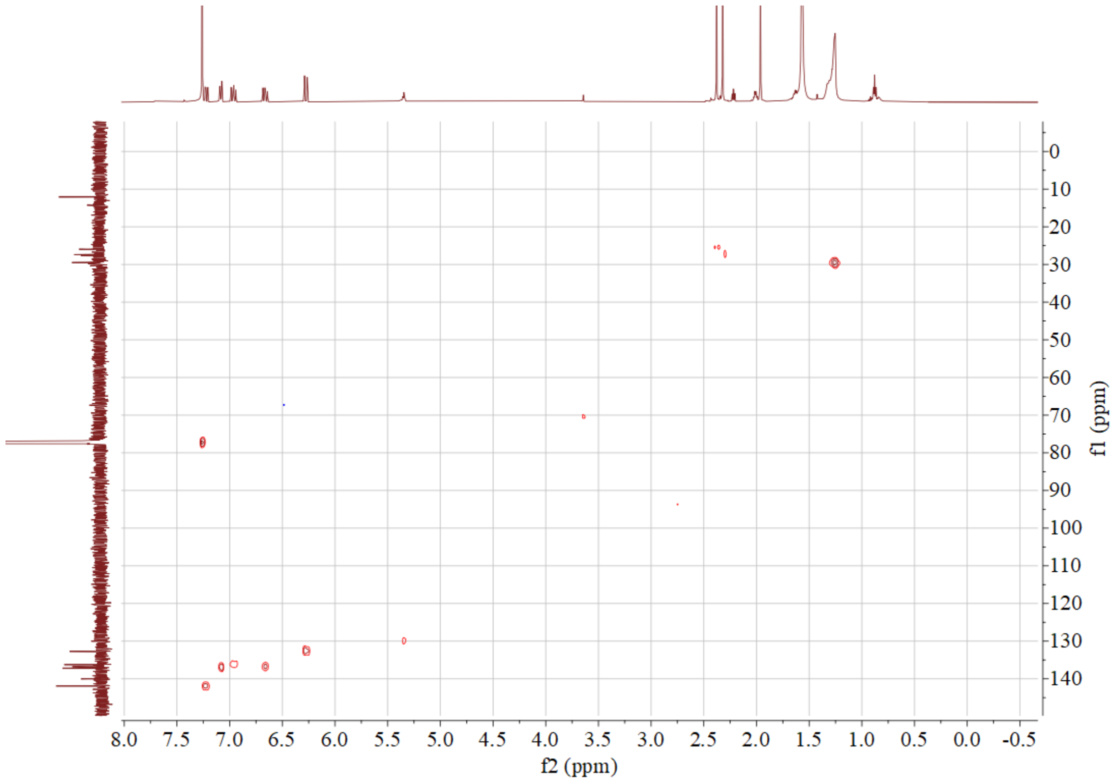

(e)

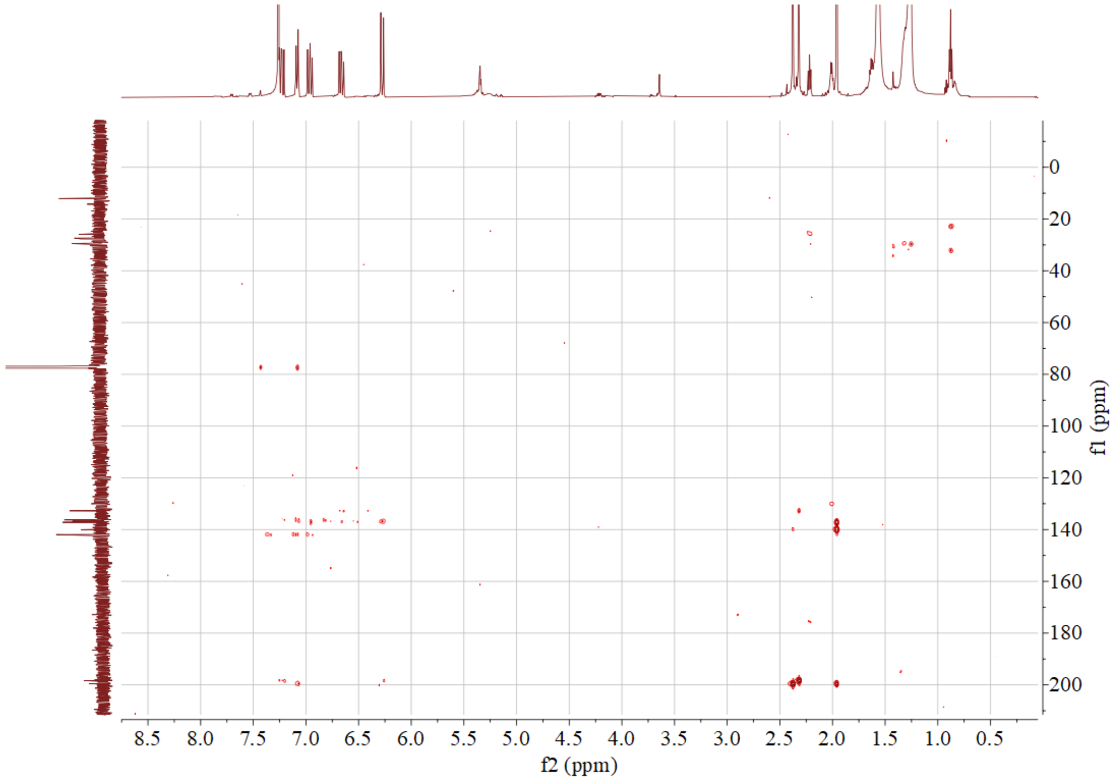

(f)

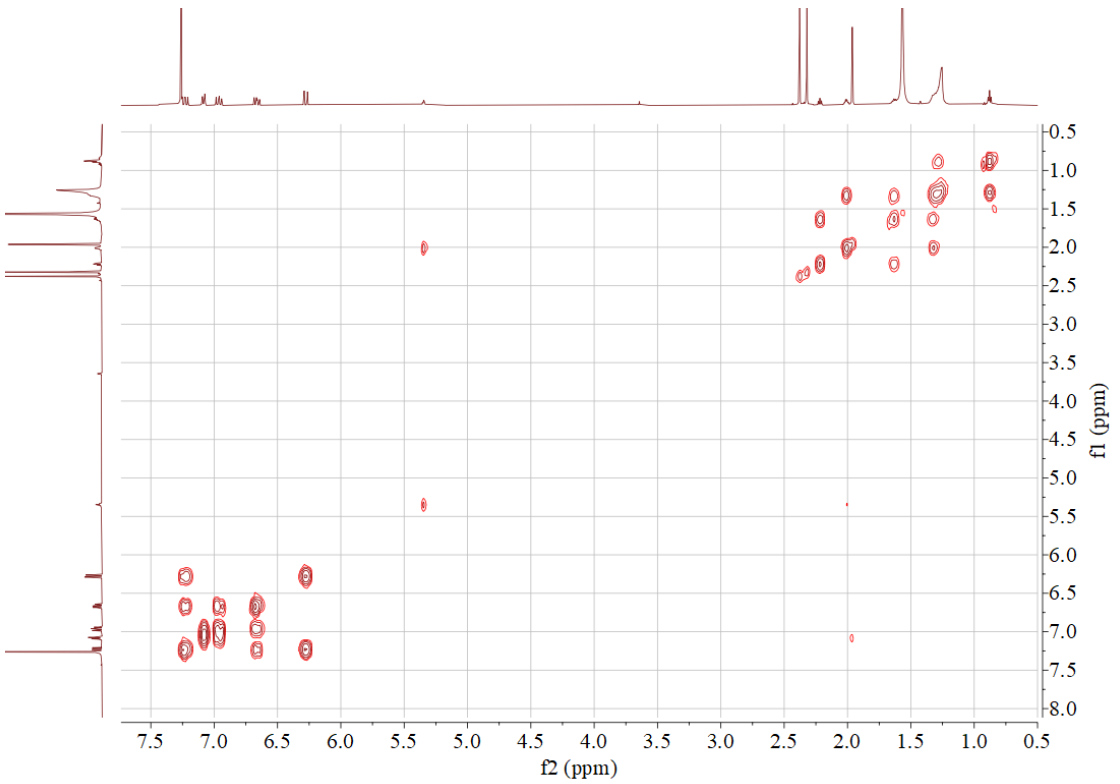

(g)

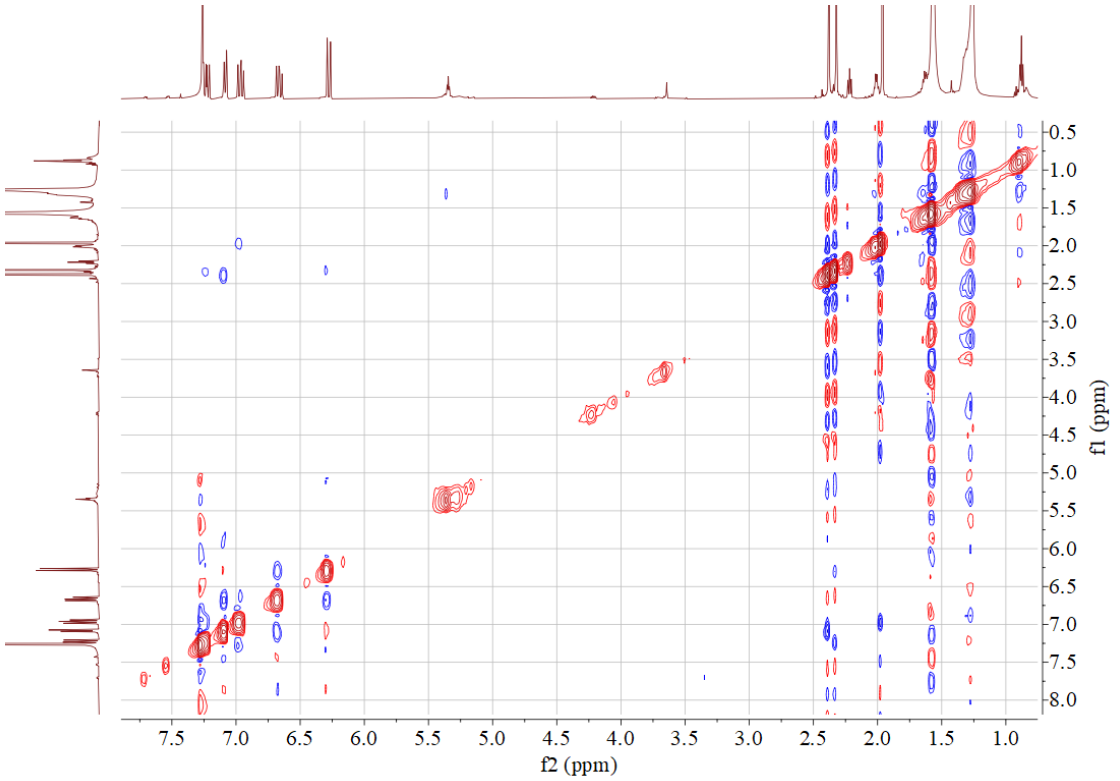

Figure S3. NMR data of (3*E*,5*E*,7*E*)-3-methyldeca-3,5,7-triene-2,9-dione (**3**). (a) Summary of the NMR data for **3**. (b) ^{1}H NMR spectrum of **3**. (c) ^{13}C NMR spectrum of **3**. (d) HSQC spectrum of **3**. (e) HMBC spectrum of **3**. (f) ^{1}H-^{1}H-COSY spectrum of **3**. (g) ROESY spectrum of **3**.

Figure S4. NMR data of **4**

(a)

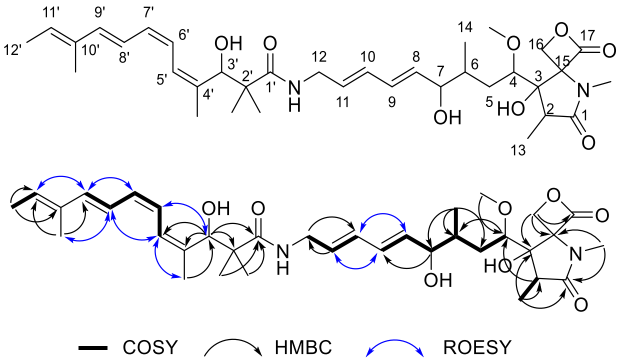

^{1}H and ^{13}C NMR data of **4** (600 MHz, 150 MHz, $CDCl_3$)

Position	δ_C	δ_H(J in Hz)
1	175	
2	42.43	2.47(q, 7.3)
3	79.75	
4	82.57	3.59(t, 4.6)
5	33.05	2.04(m), 1.39(m)
6	37.29	1.8(m)
7	77.1	3.95(m)
8	134.14	5.68(m)
9	131.37	6.2(m)
10	131.66	6.2(m)
11	129.9	5.68(m)
12	41.34	3.91(m)
13	10.27	1.22(d, 7.3)
14	17.62	0.98(d, 7.9)
15	86.13	
16	65.77	4.71(d, 6.4), 4.38(d, 6.4)
17	170.33	
1'	178.25	
2'	44.85	
2'-Me	21.68	1.1(s)
2'-Me	26.31	1.35(s)
3'	75.82	4.65(s)
4'	137.34	
4'-Me	19.62	1.8(s)
5'	125.55	6.49(d, 11.8)
6'	122.79	6.14(m)
7'	130.06	6.00(m)
8'	120.85	6.57(dd, 11.3, 15.2)
9'	139.36	6.27(d, 15.2)
10'	135.09	

10'-Me	12.24	1.8(s)
11'	128.86	5.63(d, 7.2)
12'	14.29	1.76(d, 7.1)
N-Me	26.31	2.91(s)
O-Me	57.24	3.39(s)

(b)

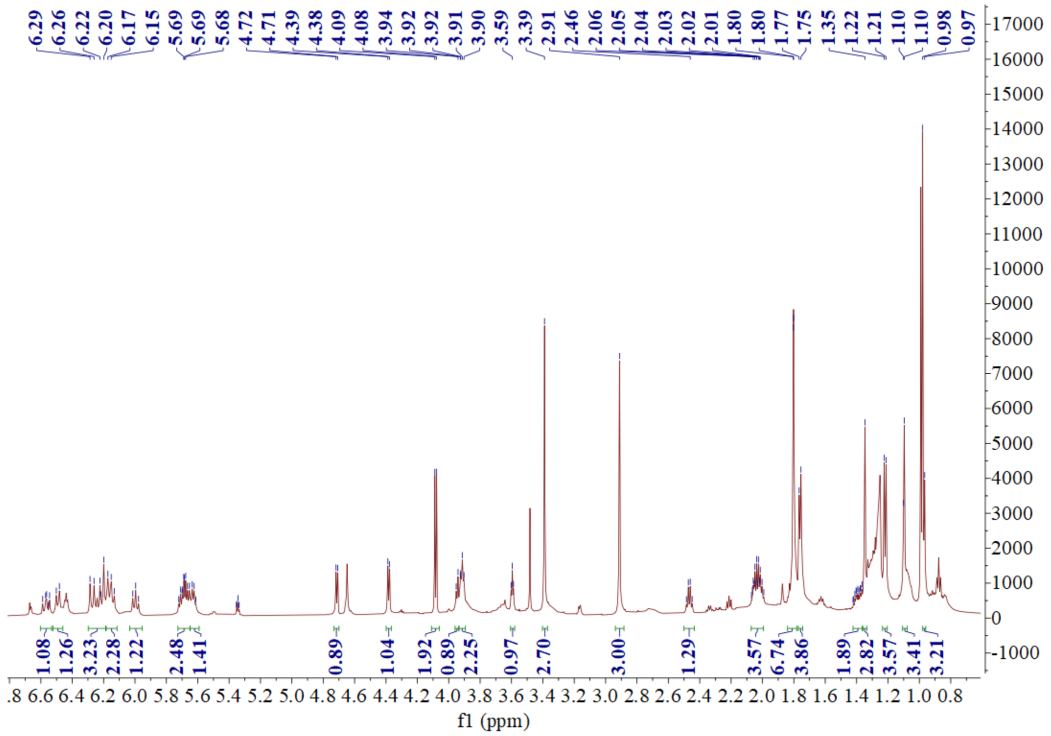

(c)

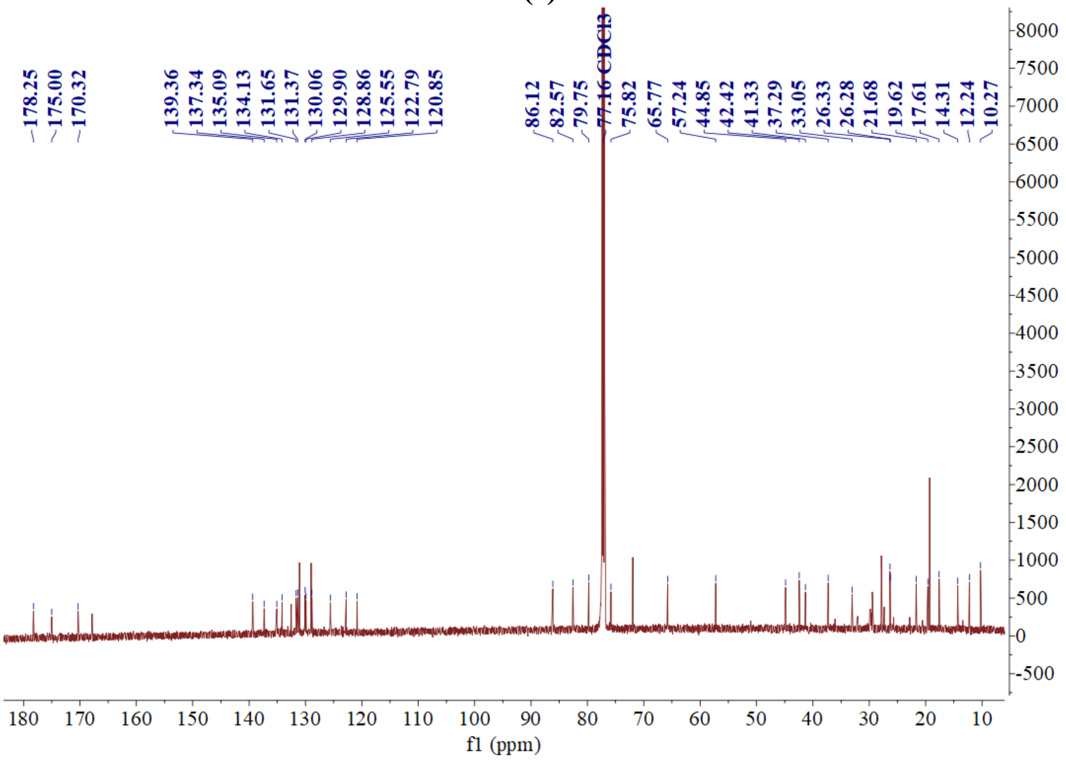

(d)

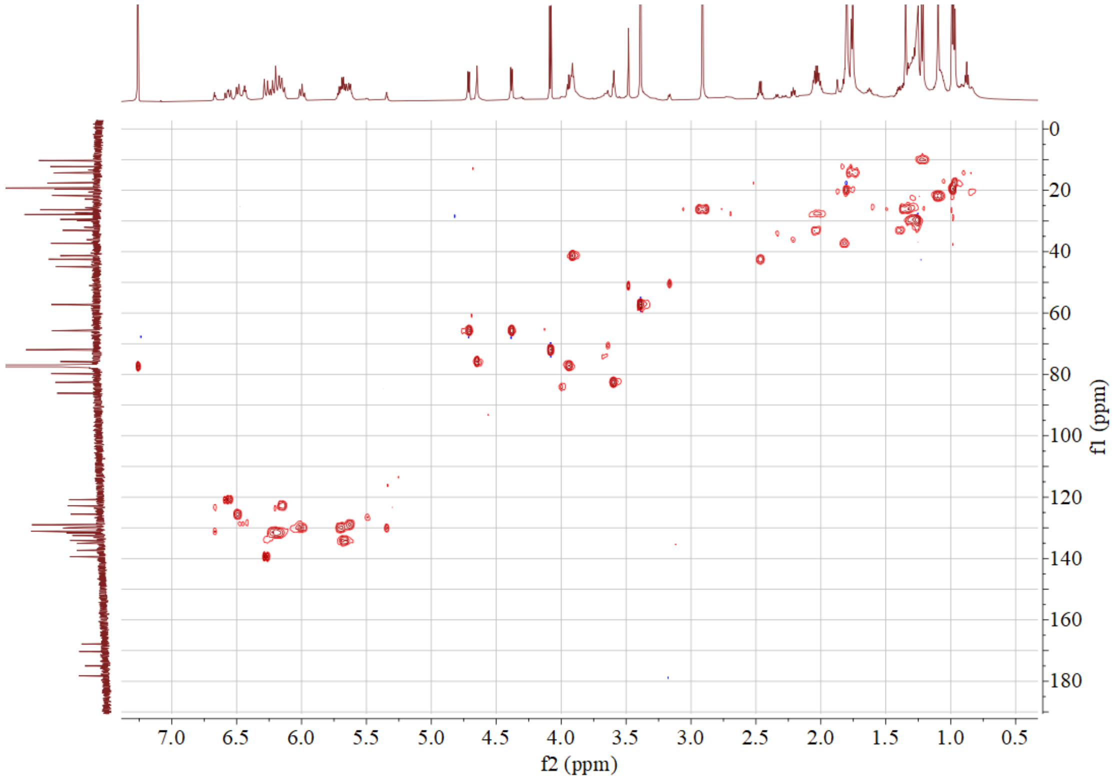

(e)

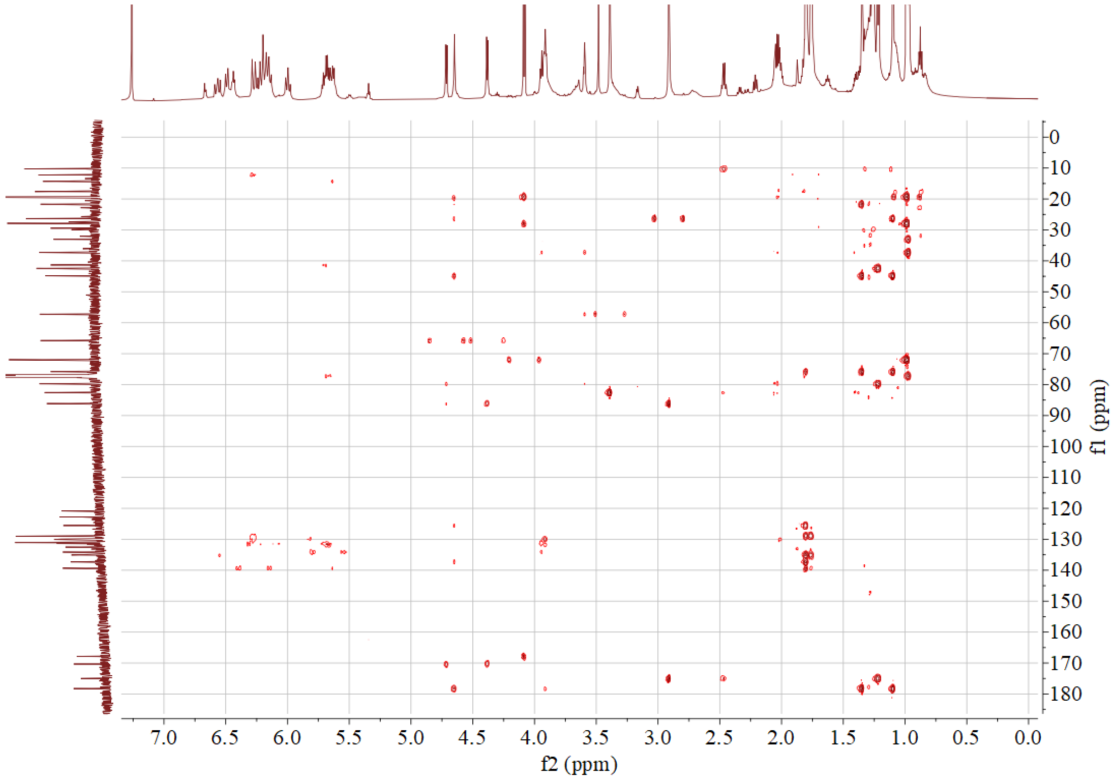

(f)

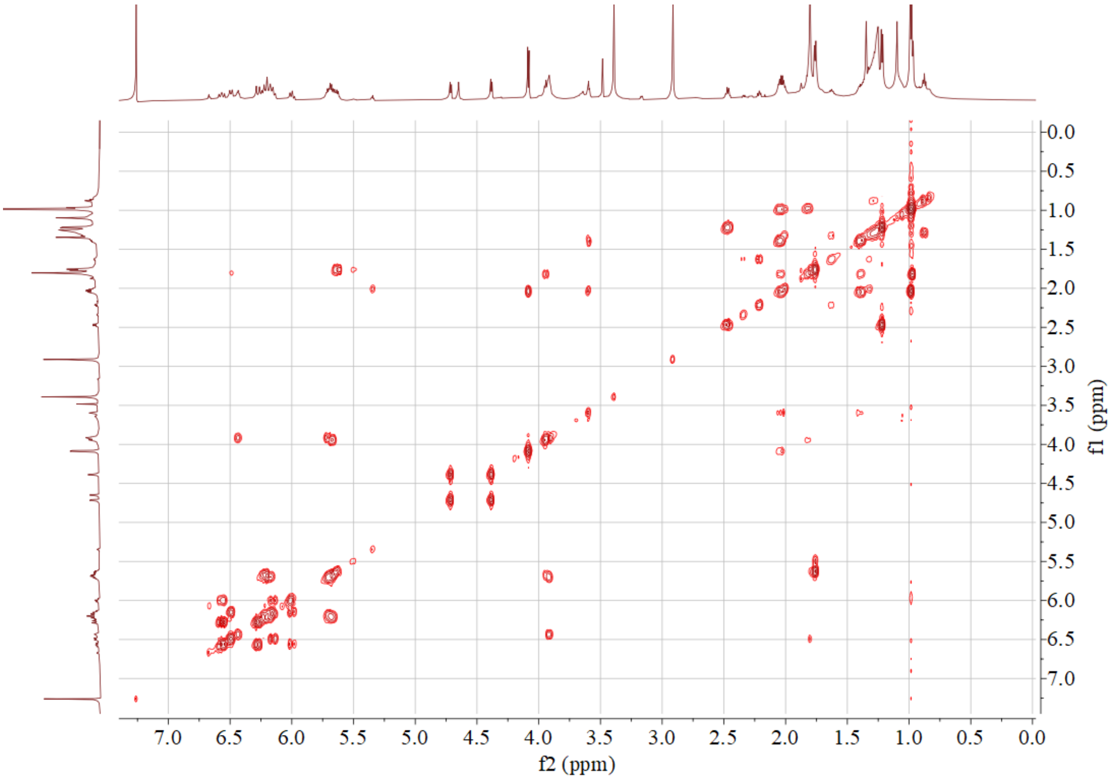

(g)

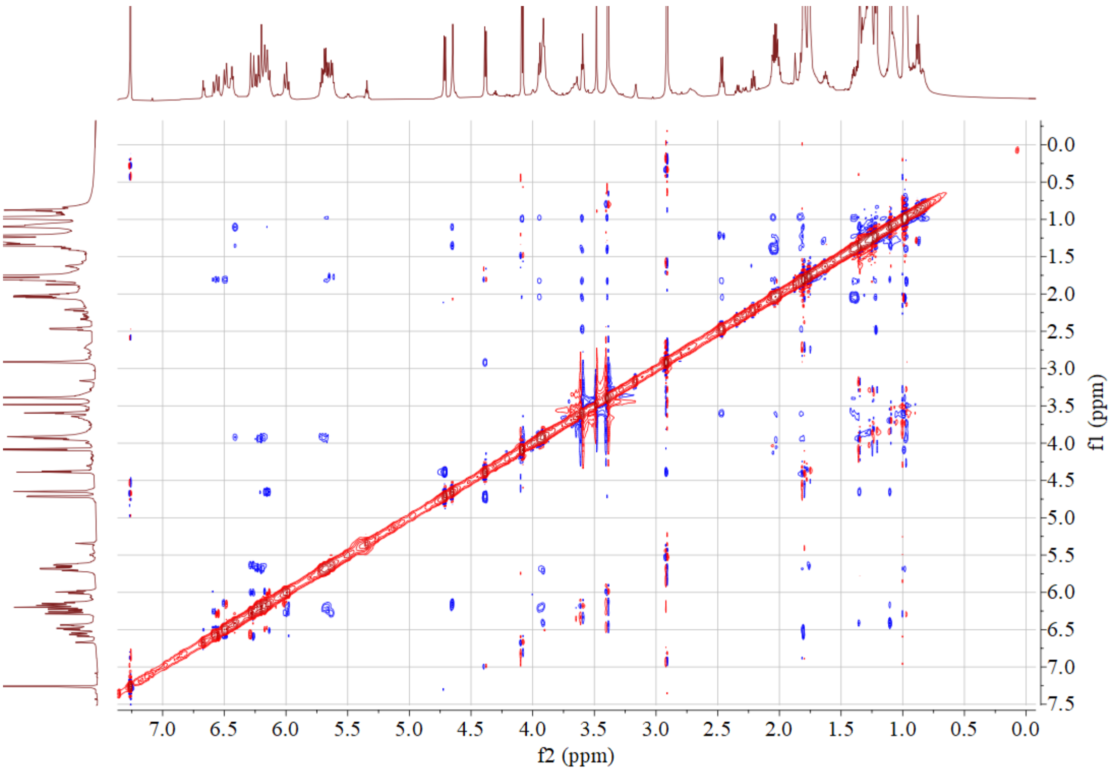

Figure S4. NMR data of denitrolajollamycin(**4**). (a) Summary of the NMR data for **4**. (b) ^{1}H NMR spectrum of **4**. (c) ^{13}C NMR spectrum of **4**. (d) HSQC spectrum of **4**. (e) HMBC spectrum of **4**. (f) ^{1}H-^{1}H-COSY spectrum of **4**. (g) ROESY spectrum of **4**.

Figure S5

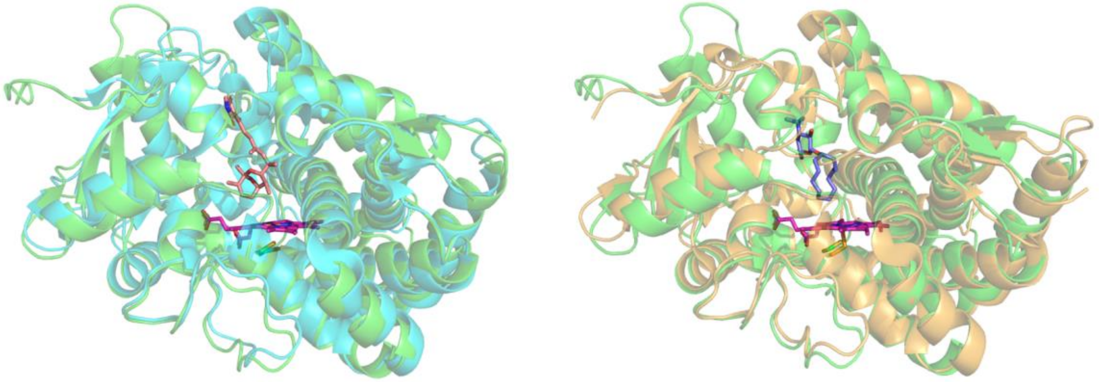

Figure S5. Superposition of the AlphaFold-predicted structure of Laj2 with the crystal structures of TamI (PDB: 6XA2) and PikC (PDB: 2WHW). Laj2 is shown in green, TamI is shown in cyan (left panel), PikC is shown in orange (right panel). Heme cofactor and the ligands co-crystalized with TamI or PikC are also displayed.

Figure S6.

(a)

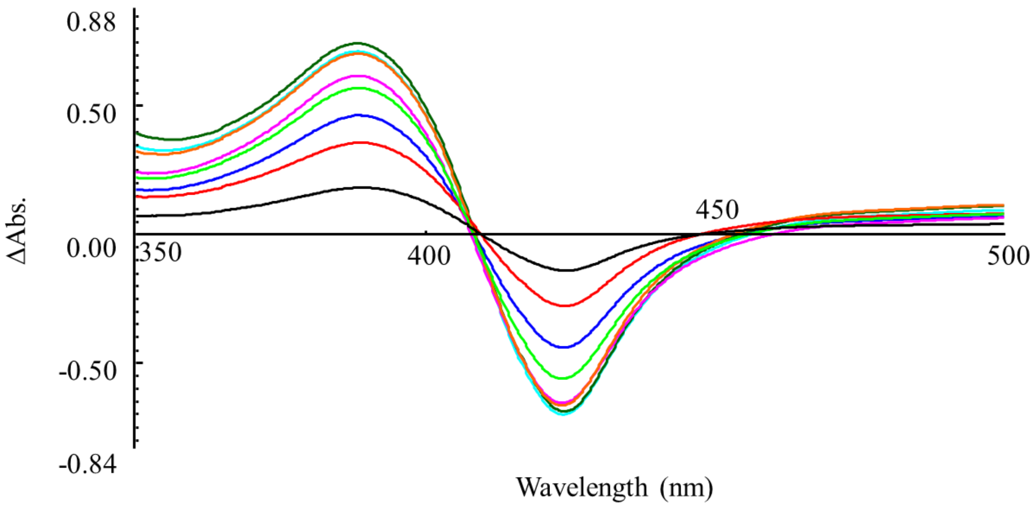

(b)

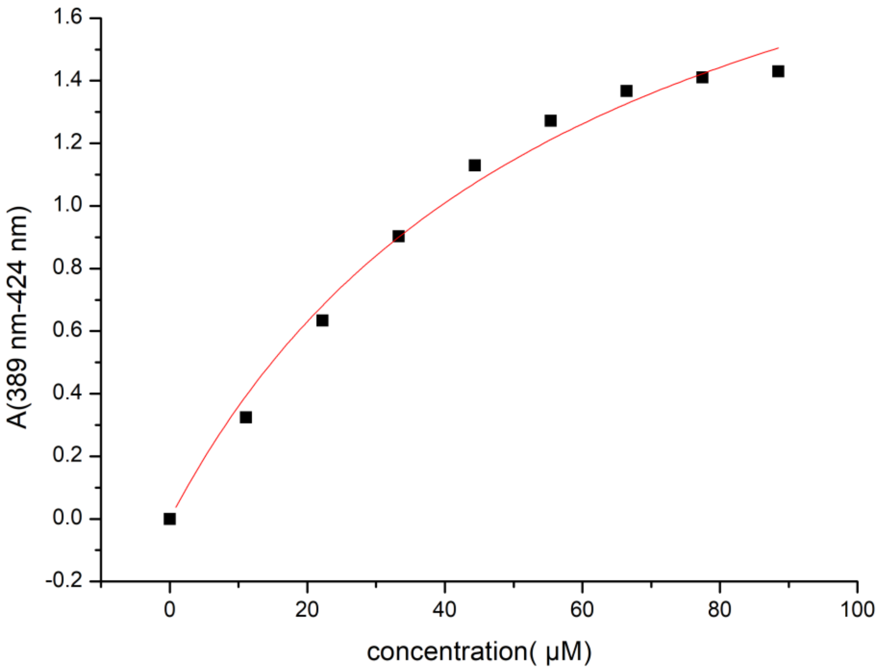

Figure S6. Analysis of denitrolajollamycin (**4**) binding to Laj2 by UV-Vis spectroscopy. (a) Type I spectra from titration of Laj2 (14 μM) with denitrolajollamycin (**4**) (0 to 88 μM), λ_{max} 389 nm, λ_{min} 424 nm. (b) The plot shows the difference in absorbance at 389 nm and 424 nm versus denitrolajollamycin (**4**) concentration. The dissociation constant for Laj2 was estimated as ~60 μM.

Figure S7. UV-Vis spectrums of Lajollamycin

(a)

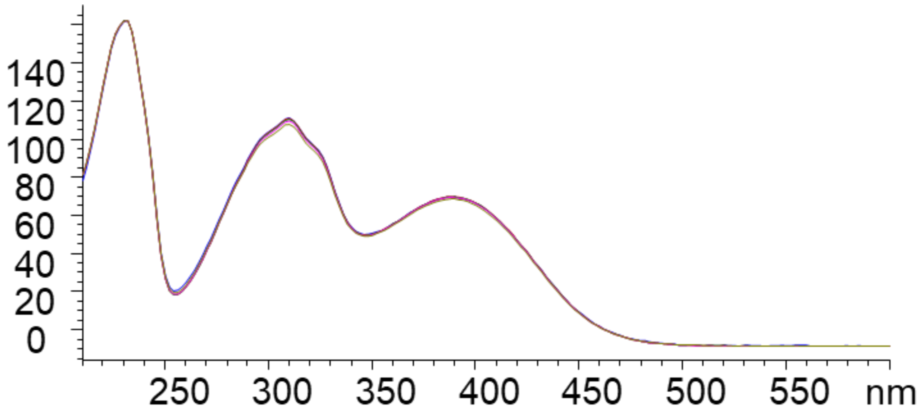

(b)

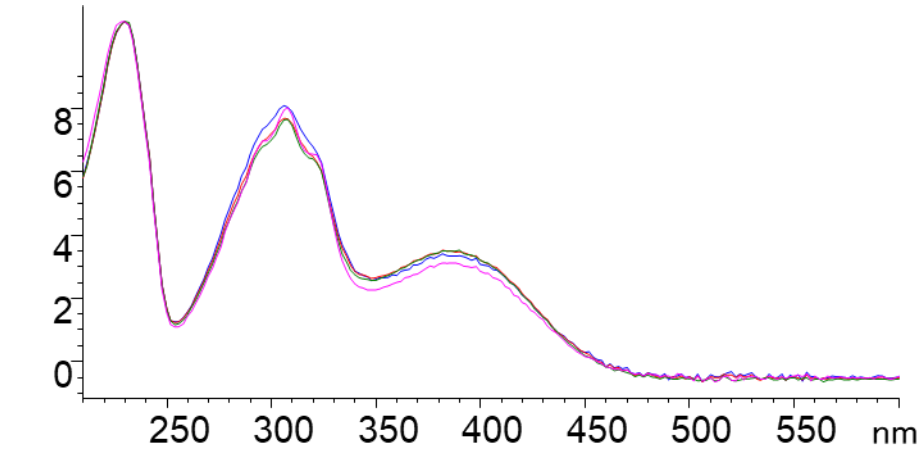

Figure S7. Comparison of UV-Vis spectra of lajollamycin (**1b**) and the product from the in vitro reaction of Laj2. (a) The UV-Vis spectrum of lajollamycin (**1b**). (b) The UV-Vis spectrum of the product from the in vitro reaction of Laj2.

Reference

[1] Sun Y, Ma L, Han D, Du L, Qi F, Zhang W, et al. In vitro reconstitution of the cyclosporine specific P450 hydroxylases using heterologous redox partner proteins. J Ind Microbiol Biotechnol 2017; 44:161–6.

[2] Schallmey A, den Besten G, Teune IGP, Kembaren RF, Janssen DB. Characterization of cytochrome P450 monooxygenase CYP154H1 from the thermophilic soil bacterium Thermobifida fusca. Appl Microbiol Biotechnol 2011; 89:1475–85.

[3] Li S, Podust LM, Sherman DH. Engineering and Analysis of a Self-Sufficient Biosynthetic Cytochrome P450 PikC Fused to the RhFRED Reductase Domain. J Am Chem Soc 2007; 129:12940–1.
